# Targeting Oncogenic Super Enhancers in *MYC-*Dependent AML Using a Small Molecule Activator of NR4A Nuclear Receptors

**DOI:** 10.1038/s41598-020-59469-3

**Published:** 2020-02-18

**Authors:** S. Greg Call, Ryan P. Duren, Anil K. Panigrahi, Loc Nguyen, Pablo R. Freire, Sandra L. Grimm, Cristian Coarfa, Orla M. Conneely

**Affiliations:** 1 Department of Molecular and Cellular Biology, Baylor College of Medicine, Houston, TX 77030 USA; 2 Molecular and Cellular Biology PhD Program, Baylor College of Medicine, Houston, TX 77030 USA; 3 Integrative Molecular and Biomedical Sciences PhD Program, Baylor College of Medicine, Houston, TX 77030 USA; 4 Dan L Duncan Comprehensive Cancer Center, Baylor College of Medicine, Houston, TX 77030 USA; 5 Center for Precision Environmental Health, Baylor College of Medicine, Houston, TX 77030 USA

**Keywords:** Acute myeloid leukaemia, Oncogenes, Cancer epigenetics, Targeted therapies

## Abstract

Epigenetic reprogramming in Acute Myeloid Leukemia (AML) leads to the aberrant activation of super enhancer (SE) landscapes that drive the expression of key oncogenes, including the oncogenic *MYC* pathway. These SEs have been identified as promising therapeutic targets, and have given rise to a new class of drugs, including BET protein inhibitors, which center on targeting SE activity. NR4A nuclear receptors are tumor suppressors of AML that function in part through transcriptional repression of the *MYC*-driven oncogenic program via mechanisms that remain unclear. Here we show that NR4A1, and the NR4A inducing drug dihydroergotamine (DHE), regulate overlapping gene expression programs in AML and repress transcription of a subset of SE-associated leukemic oncogenes, including *MYC*. NR4As interact with an AML-selective SE cluster that governs *MYC* transcription and decommissions its activation status by dismissing essential SE-bound coactivators including BRD4, Mediator and p300, leading to loss of p300-dependent H3K27 acetylation and Pol 2-dependent eRNA transcription. DHE shows similar efficacy to the BET inhibitor JQ1 at repressing SE-dependent *MYC* expression and AML growth in mouse xenografts. Thus, DHE induction of NR4As provides an alternative strategy to BET inhibitors to target *MYC* dependencies via suppression of the AML-selective SE governing *MYC* expression.

## Introduction

Transcriptional regulation of hematopoietic cell development is dependent on the coordination between master transcription factors, transcriptional coregulators, and epigenetic machinery, which work together to influence chromatin accessibility and gene expression^1–3^. Mutations affecting these factors contribute to the aberrant reprogramming of enhancer landscapes, resulting in abnormal enhancer-regulated gene expression, and eventual leukemic transformation^4–10^. Recent findings have highlighted a subset of highly active super-enhancers (SEs) consisting of enhancer clusters spaced over large genomic regions that function cooperatively to maintain robust gene transcription. SEs are distinguished from typical enhancers by exceptionally high enrichment of master transcription factors, transcriptional coactivators, including p300, BRD4 and Mediator, and the active histone mark H3K27Ac^11–14^. Active SEs are also enriched with RNA Pol II, which transcribes enhancer RNAs (eRNAs) that functionally contribute to target gene expression^15–17^. In normal physiology, SEs respond to developmental signaling pathways to maintain the expression of cell identity genes^11,12,18^ and regulate hematopoietic differentiation^19^. SEs are also aberrantly activated in cancer cells, including AML, to promote the expression of oncogenic drivers of malignancy^11,18,20^.

The *c-MYC* proto-oncogene (hereafter referred to as *MYC*) is a common driver of leukemogenicity and AML progression^21^. *MYC* overexpression occurs in a broad range of cytogenetically distinct AMLs and is associated with poor overall survival^22^. *MYC* plays a key role in AML maintenance where it contributes to enhanced RNA biogenesis and translation, cell growth, leukemia stem cell self-renewal, and resistance to chemotherapy^23–27^. Because of the central role of *MYC* as a key oncogenic driver of a spectrum of cytogenetically distinct AMLs, targeting *MYC* is a key objective in the development of new targeted AML therapeutics. To this end, an AML-selective distal super enhancer (SE) thought to govern *MYC* expression was recently described and represents a novel epigenetic vulnerability for the development of therapies targeting *MYC*^5,19,28^. Current strategies have focused primarily on epigenetic drug targeting of chromatin factors required for maintaining SE activation (BRD4, p300, Mediator)^20,28–31^. In contrast, relatively little is known regarding the identity of factors that repress SE activation. Further, because of the global epigenetic effects of chromatin factors on transcriptional regulation, more selective targets are warranted.

Orphan nuclear receptors NR4A1 and NR4A3 are potent functionally-redundant tumor suppressors of AML that are frequently silenced across human AMLs through blockade of transcription elongation^32,33^. Forced expression of NR4A1 represses AML cell viability via inhibition of AML cell proliferation both *in vitro* and *in vivo*, and results in strong suppression of the oncogenic *MYC* pathway in t(8,21) rearranged human AML cells^34^. Using in silico chemical genomics screening, we recently identified the FDA-approved drug dihydroergotamine (DHE) as a small molecule inducer of silenced NR4As, which promotes NR4A-dependent suppression of AML cell proliferation and exhibits antileukemic activity across a subset of cytogenetically distinct human AML cells both *in vitro* and in xenograft models of human AML^33^.

In the current study, we address the global NR4A dependent mechanisms of DHE action in DHE sensitive MLL-rearranged human AML cells. We show that DHE regulates overlapping target genes with NR4A1, including repression of a select group of AML oncogenes, by decommissioning a subset of NR4A-bound SEs, including the *MYC* SE. We show that NR4A1 binds directly to the *MYC* SE where it dismisses essential coactivators, leading to loss of SE functional activity by eliminating Pol II-dependent eRNA transcription and enhancer-promoter looping. Finally, we show that the efficacy of DHE in suppressing SE-dependent expression of *MYC in vitro*, and *MYC*-dependent AML maintenance *in vivo*, is similar to that of BET bromodomain inhibitor JQ1. These data predict that DHE reactivation of NR4A nuclear receptors provides an alternative strategy to BET inhibitors to target *MYC* dependencies in AML cells via suppression of the AML-selective SE governing *MYC* expression.

## Materials and Methods

### Human leukemic cell lines

Human leukemic cell lines (MOLM-14, MV4–11, K562, Kasumi-1) were purchased from ATCC, or obtained from collaborators at Baylor, and validated by the Baylor Tissue Culture Core. Lines were cultured according to the protocols outlined by the ATCC and DSMZ. For cell viability assays, 10^5^ cells were plated at day zero and exposed to treatment for 96 hours. Cell counts were measured every 24 hours using trypan blue staining and a hemacytometer.

### Real-time quantitative polymerase chain reaction (RT-qPCR)

For gene expression measurements, 1 × 10^6^ cells were used for experimental replicates. RNA was extracted from cells using Qiashredder columns and RNeasy kits (Qiagen). Extracted RNA was measured using a BioPhotometer spectrophotometer (Eppendorf). Reverse transcription of RNA molecules was done with a High Capacity cDNA Reverse Transcription Kit (Applied Biosystems), using 1ug RNA input from each sample. Samples were diluted 1:5 with H_2_O and qPCR was prepared using Taqman Gene Expression Master Mix (Applied Biosystems) and run using ABI Step One Plus Sequence Detection System (Applied Biosystems). Taqman probes include NR4A1 (Hs00374230_m1), NR4A3 (Hs00545007_m1), MYC (Hs00153408_m1), BRD4 (Hs04188087_m1), MED1 (Hs01062349_m1), and B2M (Hs00984230_m1). Expression was calculated using ΔΔCt, and samples were normalized to house-keeping gene B2M. Error bars represent standard deviation, and p-values were calculated using two-tailed student’s t-test with statistical significance at p < 0.05.

### ***In vitro*** transcription

*In vitro* transcribed RNA was generated using an mMESSAGE mMACHINE T7 Ultra kit (Thermo Fisher Scientific) with linearized pcDNA3.1 plasmids containing GFP or NR4A1 as template DNA. IVT RNA was polyadenylated using a Poly(A) Tailing Kit (Applied Biosystems), and purified using a MEGA Clearance Kit (Applied Biosystems). Cell lines were combined with IVT RNA at a final concentration of 100 nM in 0.4 cm cuvettes (USA Scientific) and electroporated at 330 V for 5 ms using the GenePulser Xcell electroporation system (Bio-Rad).

### siRNA knockdown

For siRNA knockdown of BRD4 or MED1, MOLM-14 cells were electroporated with 300 nM of siRNA targeting either BRD4 (Dharmacon), MED1 (Dharmacon), or a non-targeting control (Sigma). Electroporation of siRNA was done at −48 hours, and again at −24 hours prior to RNA extraction. 48 hours following initial electroporation, cells were counted and partitioned into experimental replicates for RNA extraction and RT-qPCR.

### Chromatin immunoprecipitation (ChIP)

Cells for ChIP were cross-linked using an 11% formaldehyde solution (50 mM HEPES-KOH pH 7.5, 100 nM NaCl, 1 mM EDTA, 0.5 mM EGTA, 11% formaldehyde) for 10 minutes, followed by quenching with glycine at 125 mM for 5 minutes. Pellets were washed and centrifuged twice at 4 °C, followed by snap freezing in liquid nitrogen. For all buffers involving cell lysis, sonication, IP, or washing, fresh protease and phosphatase inhibitors were included at 1X concentration (Thermo Fischer Scientific). Cell pellets were resuspended in Lysis Buffer 1 (50 mM HEPES-KOH pH 7.5, 140 mM NaCl, 1 mM EDTA, 10% Glycerol, 0.5% NP-40, 0.25% Triton X-100) and rotated at 4 °C for 10 minutes, followed by a 5 minute centrifugation at 1400 g. Cell pellet was resuspended in Lysis Buffer 2 (10 mM Tris-HCl pH 8.0, 200 mM NaCl, 1 mM EDTA, 0.5 mM EGTA), rotated for 10 minutes at 4 °C and centrifuged for 5 minutes at 1400 g. Cell pellet was then resuspended in either Histone Sonication Buffer (10 mM Tris-HCl pH 8.0, 100 mM NaCl, 1 mM EDTA pH 8.0, 0.5 mM EGTA, 0.1% Sodium Deoxycholate, 0.2% SDS), or TF Sonication Buffer (50 mM Tris-HCl pH 7.5, 140 mM NaCl, 1 mM EDTA, 1 mM EGTA, 0.1% SDS, 1% Triton X-100) as appropriate. Sonication was performed with the BioRuptor water bath sonicator (Diagenode) using 30 seconds on/30 seconds off cycles. Cells were sonicated for 30 cycles (30 minutes) and cellular debris was removed via centrifugation. Experimental replicates were then aliquoted for IPs for ChIP, each IP using the lysate from approximately 2.5 × 10^6^ cells. Inputs were pooled among each experimental condition into single inputs and then incubated at 65 °C overnight to reverse the crosslinks. IP aliquots were diluted 1:10 with ChIP dilution buffer (1% Triton X-100, 150 mM NaCl, 2 mM EDTA, 20 mM Tris-HCl pH 8.0) and then incubated overnight with rotation at 4 °C with 2ug of the appropriate antibody. 30 uL magnetic protein g Dynabeads (Thermo Fisher Scientific) were added to each sample and then incubated for 3hrs with rotation at 4 °C. Using a magnetic tube rack (Invitrogen), the supernatant was removed and discarded and magnetic beads were washed 1x with low salt wash buffer (1% Triton X-100, 150 mM NaCl, 2 mM EDTA, 20 mM Tris-HCl pH 8.0), 1x with high salt wash buffer (1% Triton X-100, 500 mM NaCl, 2 mM EDTA, 20 mM Tris-HCl pH 8.0), 1x LiCl wash buffer (0.25 M LiCl, 1% NP-40, 1% deoxycholate, 1 mM EDTA, 10 mM EDTA, 10 mM Tris-HCl pH 8.0), and 3x TE Buffer (2 mM EDTA, 20 mM Tris-HCl pH 8.0). LiCl wash was omitted for IPs using Bethyl antibodies. For each wash, beads were incubated with wash buffer for 5 minutes at 4 °C with rotation. Following the final wash, beads were resuspended in 120 uL freshly-prepared elution buffer (1% SDS, 100 mM NaHCO_3_) and incubated at 65 °C overnight to reverse the crosslinks. Samples were then combined with RNase A (Thermo Fisher) at a final concentration of 0.2 mg/mL and incubated at 37 °C for 2 hours. Following RNase digestion, samples were combined with proteinase K (Sigma Aldrich) at a final concentration of 0.2 mg/mL and incubated at 55 °C for 2 hours. Following proteinase K digestion, inputs and ChIP samples were purified using a PCR Purification Kit (Qiagen) with a final elution of 120 uL. Standard curve for qPCR was made by pooling aliquots of each input and performing 5-fold serial dilutions. Samples were quantified with qPCR using POWERUP SYBR Green Master Mix (Fisher Scientific) and run using ABI Step One Plus Sequence Detection System (Applied Biosystems). ChIP results are represented as percentage of input based on quantity means derived from a standard curve. In the case of histone ChIPs, the enrichment is represented as a value normalized to total histone (H3). Error bars represent standard deviation, and p-values were calculated using two-tailed student’s t-test with statistical significance at p < 0.05. Primer sequences for ChIP experiments are indicated in Supplemental Table: ChIP-qPCR Primer Sequences. ChIP antibodies include RNA Pol II CTD (Abcam), Pol II Phospho Serine 5 (Abcam), Pol II Phospho Serine 2 (Abcam), Histone H3 (Abcam), Histone H3K27Ac (Abcam), Histone H3k36me3 (Abcam), NR4A (pan NR4A antibody, Santa Cruz), CDK9 (Santa Cruz), CDK8 (Bethyl), MED1 (Bethyl), p300 (Bethyl), and BRD4 (Bethyl). Antibody identifiers are listed in the Supplemental Resource Table.

### 3C (Chromatin conformation capture) assays

Treated cells were cross-linked with 1% formaldehyde and quenched with 0.125 M glycine. Pelleted cells were washed with PBS and incubated in 1 mL Cell Lysis Buffer (10 mMTris-HCl pH 7.5, 10mM NaCl, 0.2% Igepal CA-630; supplemented with 1x protease inhibitor cocktail [Roche], and 0.2 mM PMSF) on ice for 20 minutes. Cell pellets were washed 3x in 1 mL cold Cell Lysis Buffer, and 2x in 1.2x NEB3.1 Buffer (0.2 mL). Cells were resuspended in 0.2 mL 1.2x NEB3.1, SDS was added to a final concentration of 0.3%, and samples were incubated at 37 °C for 1 hour. Triton X-100 was added to a final concentration of 1% and samples were incubated at 37 °C for 1 hour. Samples were then digested with 15 uL of BglII (150 U, New England Biolabs) at 37 °C overnight with gentle mixing. The following day, SDS was added to a final concentration of 0.6%, and the samples were incubated at 65 °C for 30 minutes. Samples were then diluted with 1.4 mL 1.15x T4 DNA Ligation Buffer (New England Biolabs) with 1% Triton X-100, and incubated at 37 °C for 1 hour. DNA ends were then ligated with 400 U of T4 DNA Ligase (New England Biolabs) and incubated at 16 °C for 4 hours. The samples were de-proteinized and de-crosslinked by incubating at 65 °C overnight with 10 uL (100ug) Proteinase K (Sigma Aldrich). DNA was extracted with phenol-chloroform, precipitated with ethanol, and dissolved in 100 uL H_2_O. Samples aliquoted prior to BglII digestion, after digestion, and after ligation were analyzed by PCR to monitor the digestion and ligation efficiencies. The ligation detection and quantification of cross-linking efficiency involved 2 rounds of PCR: regular PCR in round 1, followed by qPCR in round 2, using nested primers (For primer sequences, see Supplemental Table: 3C Chromatin Looping qPCR Primer Sequences). One ug of DNA was used per sample in the 1st round of PCR using One Taq Hot-Start DNA Polymerase in a 20 uL reaction (New England Biolabs). One uL of round 1 PCR product was used in each qPCR reaction in a total volume of 15 uL reaction using POWERUP SYBR Green Master Mix (Fisher Scientific). Each round 1 PCR was followed up by triplicate qPCR reactions.

Recombinant BAC clones were obtained from BAC PAC Resources. An equimolar mixture of BAC clones RP11-770K21 (Encompasses the *MYC* super enhancer) and CTD-2034C18 (Encompasses the *MYC* locus) was digested with BglII, precipitated, and ligated with T4 DNA Ligase; this “cut-and-ligated BAC DNA” served as a source for inter-fragment ligants that represent a theoretical optimum for ligant abundance. Ten ng of this “normalizer” ligant population was subjected to PCR and qPCR alongside the samples. We subtracted the “normalizer” C_T_ values for each primer pair from the corresponding C_T_ values obtained from the samples. This subtraction (ΔC_T_) for each amplicon (each primer pair/ligant) was used to calculate the relative ligant abundance (2∧^−ΔCT^), which is same as contact efficiency. This value is presented here as “Looping Index^3C^”.

### Enhancer RNA (eRNA) measurement

RNA was extracted from samples using the Qiashreddar and RNeasy kits as previously described. Residual genomic DNA was digested with DNase (Ambion DNA-Free DNA Removal Kit, AM1906), which was heat inactivated following incubation using DNase inactivation reagent. RNA samples were subjected to first strand cDNA synthesis using a SuperScript II kit (Invitrogen 18064-014) using random primers. The cDNA preparation was diluted 10-fold with H_2_O, and 1 uL was used in a 15 uL qPCR reaction using POWERUP SYBR Green Master Mix (Fisher Scientific), which was plated in triplicate for each biological replicate using primers specific to each eRNA (See Supplemental Table: Enhancer RNA qPCR Primer Sequences). The relative RNA levels were normalized to ACTB transcript levels.

### CRISPR interference assay

*MYC* super enhancer interference was achieved using the lentiviral plasmid pLV hU6-sgRNA hUbC-dCas9-KRAB-T2a-Puro (Addgene# 71236), with cloned guide RNAs targeting the E5 enhancer or a non-targeting control (See Supplemental Table: CRISPR Interference Oligos). MOLM-14 cells were transduced with lentiviral constructs and assessed for *MYC* expression and cell viability.

### ChIP-Seq, RNA-Seq

RNA-Seq and ChIP-Seq datasets were deposited to the NCI Geo Database (Accession# GSE124963). For ChIP-Seq, after following the ChIP protocol previously outlined, sample library prep was done using the ThruPLEX DNA-Seq 6S Kit (Takara Bio Inc). A qPCR assay was performed on the libraries to determine the concentration of adapter ligated fragments using the Applied Biosystems ViiA 7 Quantitative PCR instrument and a Library Quantification Kit (KAPA, KK4824). All samples were pooled equimolarly and requantitated by qPCR, and also re-assessed on the Bioanalyzer. Using the concentration from the ViiA7 TM qPCR machine above, 1.8pM of equimolarly pooled library was loaded onto a NextSeq 500 High Output v2 flowcell (Illumina, FC404-2005) and amplified by bridge amplification using the Illumina NextSeq 500 sequencing instrument. PhiX Control v3 adapter-ligated library was spiked-in at 1% by weight to ensure balanced diversity and to monitor clustering and sequencing performance. A Single-end 75 cycle run was used to sequence the flowcell on a NextSeq 500 Sequencing System.ChIP-Seq reads were initially trimmed with Trim Galore. ChIP-Seq reads were then aligned to the hg19 build using bowtie2, followed by peak detection using MACS2. The identification of super enhancers was performed using the ROSE algorithm. Chip-Seq signal heatmaps were generated using the HOMER software and the python scientific library. MED1 signal tracks were generated using bedtools software. Differential MED1 signal was detected using the DiffReps software, with significance achieved at q < 0.25 and fold change exceeding 2x. Analysis of NR4A overlap with MED1 super enhancers was generated using the bedtools software. For RNA-Seq, MOLM-14 cells were electroporated with equimolar concentrations (100nM) of either GFP or NR4A1 IVT RNA, or treated vehicle (DMSO) or 10uM DHE and cultured for 6 hours. For each treatment, we included duplicate samples. Following the 6 hour incubation, cell pellets were collected and RNA was extracted using Qiashreddar and RNeasy Mini kits (Qiagen) with on-column DNase treatment to eliminate DNA contamination. Purified RNA was delivered to the Genomic and RNA Profiling (GARP) Core at Baylor College of Medicine. Total RNA samples were normalized to 250ng each, based on picogreen quantitation, and ERCC RNA controls were incorporated into each sample. Samples then went through ribosomal RNA reduction and subsequently fragmented and primed with random hexamers to produce first strand cDNA. During second strand cDNA synthesis, RNA templates were removed and replaced with cDNA strands containing dUTP. The ds-cDNA was then purified using AMPure XP beads (Beckman Coulter). Libraries were created from the cDNA by attaching an adenosine to the 3’ end and ligating unique adapters to the ends. The ligated products were then amplified. The resulting libraries were quantitated using a NanoDrop spectrophotometer and fragment size assessed with the Agilent 2100 Bioanalyzer. A qPCR assay was performed on the libraries to determine the concentration of adapter ligated fragments using the Applied Biosystems ViiA 7 Quantitative PCR instrument and a Library Quantification Kit (KAPA Biosystems). All samples were pooled equimolarly and quantitated by qPCR, and also assessed on the Bioanalyzer.Using the concentration from the ViiA7 TM qPCR machine above, 1.8 pM of equimolarly pooled library was loaded onto a NextSeq High Output v2.5 flowcell (Illumina) and amplified by bridge amplification using the Illumina NextSeq 500 sequencing instrument. PhiX Control v3 adapter-ligated library was spiked-in at 1% by weight to ensure balanced diversity and to monitor clustering and sequencing performance. A paired-end 75 cycle run was used to sequence the flowcell on a NextSeq 500 Sequencing System. RNA-seq and ChIP-Seq reads were initially trimmed with Trim Galore (https://www.bioinformatics.babraham.ac.uk/index.html). RNA-Seq transcript abundance was quantified with Salmon 0.11.0 using the human Gencode v29 as a reference transcriptional database. Differential gene expression was then calculated with DESeq2. RNA-Seq heatmaps were generated using R statistical software with the ‘pheatmap’ package. RNA-Seq data was also analyzed using Gene Set Enrichment Analysis.

### AML xenograft mouse models

All animal studies and experimental protocols were approved by Baylor College of Medicine’s Institutional Animal Care and Use Committee (IACUC) and Institutional Review Board (IRB). All experimental methods were performed in accordance with the relevant national as well as Baylor College of Medicine’s guidelines and regulations. For all xenograft models, 1x10^7^ cells (in 100uL) were injected into the right flank of seven-week old female NOD.Cg-PkdcscidIlr2gtm1Wjl/SzJ (NSG) mice. Estimates of tumor volume were regularly measured 2 to 3 times a week using a digital vernier caliper. Xenograft mice were maintained until the first mouse reached the institutional limits for tumor size (>1.5cm), at which point mice across all treatment groups were sacrificed and their tumor tissues were extracted and analyzed. For histological staining of tumor tissue, primary antibodies included Ki67 (1:5000; Abcam), and c-Myc (1:80; Santa Cruz), and secondary antibodies included a biotinylated goat antirabbit (1:400; Vector Laboratories). Histological images were captured, and staining was quantified, using a Zeiss Axioskop Microscope, Olympus DP72 camera, and CellSens Standard Imaging Software.

### Statistical analysis

Statistical p-values were calculated from experimental triplicates using independent student’s *t*-test. Error bars represent standard deviation, except for *in vivo* tumor measurements which represent standard error of the mean. Additional details are in the Supplementary Materials and Methods.

## Results

### DHE regulates overlapping target genes with NR4A1 and decommissions a subset of NR4A1-bound AML super enhancers

**Figure 1:**
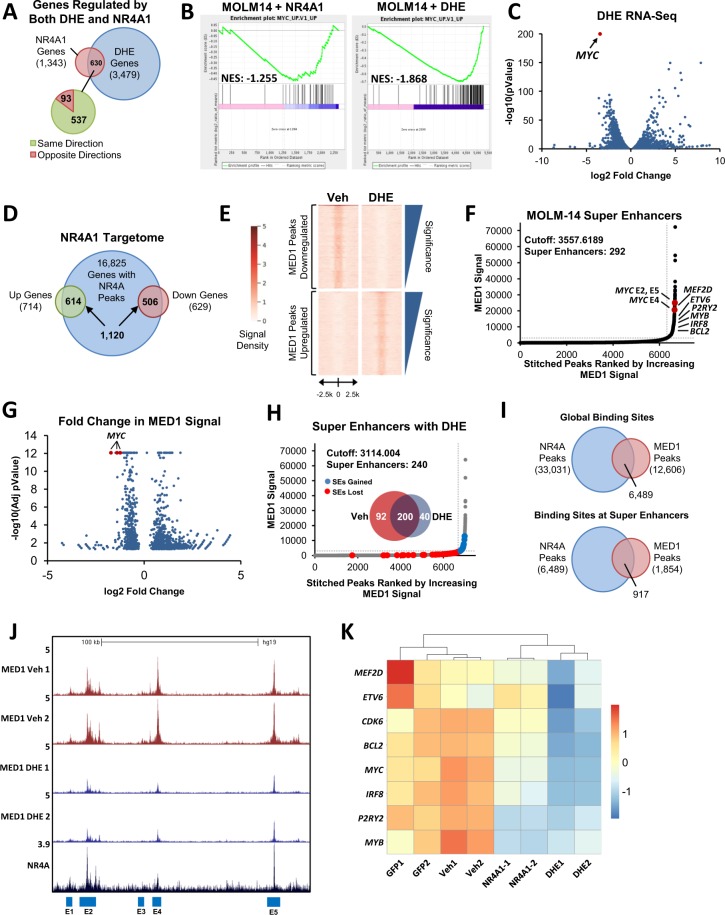
DHE Regulates Overlapping Target Genes with NR4A1 and Decommissions a Subset of NR4A1-Bound AML Super Enhancers. For analysis of acute changes in gene expression in response to NR4A1 or DHE, MOLM-14 cells were electroporated with GFP control or NR4A1 IVT RNA, or treated with vehicle or 10 uM DHE, and incubated for 6 hours. (**A**) Venn diagram summarizing gene expression overlap taken from RNA-Seq in MOLM-14 cells with NR4A1 IVT RNA expression or treatment with 10 uM DHE. (**B**) Gene set enrichment analysis reveals suppression of *MYC* pathway genes in MOLM-14 cells by NR4A1 or DHE treatment. (**C**) Volcano plot showing the distribution of genes that are upregulated or downregulated in response to DHE. Arrow points to *MYC*, which is the most statistically repressed target of DHE. (**D**) Venn diagram representing the integration of NR4A ChIP-Seq with NR4A1-dependent changes in gene expression assessed by RNA-Seq (NR4A1 targetome). (**E**) MED1 ChIP-Seq density plots comparing MED1 peaks upregulated (10,992) and downregulated (2,922) in response to DHE treatment as determined by diffReps (fold change cutoff of 2, q-val < 0.25). (**F**) ROSE analysis summary of MED1 ChIP-Seq identifying 292 super-enhancers in vehicle-treated cells. (**G**) Volcano plot summarizing fold changes in MED1 peaks in response to DHE. The *MYC* super enhancers are highlighted in red and are among the most statistically repressed MED1 peaks. (**H**) Venn diagram and ROSE summary of 240 super enhancers in DHE treated cells, highlighting super enhancers that are gained (blue) versus those that are lost (red). (**I**) Venn diagram highlighting NR4A ChIP-Seq overlap with MED1 ChIP-Seq occupancy, both on a global scale and selectively at super enhancer regions. (**J**) UCSC Genome Browser ChIP-Seq screenshot showing suppression of MED1 occupancy at the *MYC* SE region in response to DHE treatment. NR4A peaks are also displayed to show peak overlap with super enhancers. (**K**) Heatmap depiction of select SE-associated leukemic genes whose expression is repressed by both NR4A1 and DHE.

Using a chemical genomics screening approach, we recently identified an FDA-approved drug, dihydroergotamine (DHE), that relieves NR4A silencing in AML cells by promoting transcription elongation of silenced *NR4A*s. DHE effectively suppresses the growth of a subset of cytogenetically diverse AMLs, particularly those harboring MLL translocations in an NR4A-dependent manner^33^. To understand the NR4A-dependent mechanisms of DHE action in AML cells, we performed RNA-Seq analysis in DHE-responsive MLL-rearranged MOLM-14 cells to examine the global transcriptional responses to DHE, and we integrated this data with gene expression signatures (GES) regulated by forced expression of NR4A1. We identified 1,343 genes regulated by NR4A1 and of these 630 (47%) were also regulated by DHE (Fold change >1.5, pval <0.05) (Fig. 1A, Supplemental Table 1 and Supplemental Fig. 1). Gene set enrichment analysis identified a large number of genes within the *MYC* pathway that are repressed by both DHE and NR4A1 (Fig. 1B and Supplemental Fig. 2) and *MYC* was identified as the most statistically repressed gene by DHE (Fig. 1C). Thus, DHE and NR4A1 regulate an overlapping subset of gene signatures, including downregulation of a core oncogenic *MYC* signature.

We next used ChIP-seq to examine genome wide occupancy of NR4As after DHE treatment and we integrated this data with NR4A1 regulated GES. We identified 16,825 NR4A bound genomic sites, 6.7% of which were associated with NR4A1-regulated genes (+/− 100 kb from TSS) (Fig. 1D, Supplemental Table 1). The vast majority of NR4A1-regulated genes were associated with NR4A binding in DHE treated cells (86% of upregulated and 80% of downregulated genes respectively, Fig. 1D). Thus DHE dependent induction of NR4A expression leads to NR4A recruitment to the majority of NR4A regulated genes. We next sought to determine how changes in NR4A dependent gene expression in response to DHE are connected with the activity of global NR4A-bound enhancers, including AML super enhancers. Super enhancers have been identified by their ChIP-Seq profiles for H3K27Ac, BRD4 or Mediator using the rank ordering of super enhancers (ROSE) algorithm^12,13,30^. Mediator is highly sensitive to super-enhancer perturbation, and its enrichment strongly correlates with BRD4 and H3K27Ac at super enhancer sites^30^. To objectively identify global enhancers and super enhancers, and monitor their changes in response to DHE, we performed ChIP-Seq for the MED1 subunit of the Mediator complex in MOLM-14 cells with and without DHE (Supplemental Fig. 3). Using MACS2, we identified 12,656 and 12,606 MED1 peaks in vehicle and DHE treated cells respectively. However, analysis with diffReps showed that treatment with DHE led to widespread changes in the distribution of MED1 occupied enhancers resulting in a gain of 10,992 peaks, and a loss of 2,922 (Fig. 1E). The ROSE algorithm identified 292 super enhancers in untreated cells, including SEs associated with known AML oncogenes, *MYC*, *MEF2D*, *MYB*, *IRF8* and *BCL2* (Fig. 1F). Analysis of changes in MED1 signal after DHE exposure revealed that the *MYC* SE clustered among the most statistically repressed MED1 peaks, which is consistent with our RNA-Seq data (Fig. 1G). ROSE analysis comparisons between treatment groups identified a total of 200 super enhancers that were unaffected by DHE treatment, 92 that were repressed, and 40 that were gained (Fig. 1H). Of the 12,606 global binding sites identified for MED1 in DHE treated cells, 6,489 (51%) were co-enriched for NR4As. Interestingly, NR4As were co-enriched at 917 (49%) of the 1854 MED1 peaks identified within super enhancer regions (Fig. 1I). Representative examples of NR4A1-occupied super enhancers that are repressed by DHE are shown in Fig. 1J and Supplemental Fig. 4 and include those associated with AML oncogenes *MYC* (Fig. 1J), *CDK6 and BCL2*, as well as *IRF8*, *MEF2D*, *P2RY2* and *ETV6* (Supplemental Fig. 4). Importantly, DHE-dependent dismissal of MED1 at these super enhancers also closely correlated with transcriptional repression of their gene expression by both NR4A1 and DHE observed by RNA-Seq analysis (Fig. 1K). Representative examples of NR4A1-occupied super enhancers that were unaffected by DHE include those associated with *ATP8B4*, and *GRAP*, while examples of super enhancers that were gained include those associated with *PECAM1* and *UBAC2* (Supplemental Fig. 5). Taken together, these results demonstrate that DHE regulates occupancy of MED1 at a select subset of NR4A1-bound SEs. Among these, DHE dismisses MED1 binding at a select group of SE-associated AML oncogenes whose transcription is repressed by both NR4A1 and DHE.

### NR4A1 disrupts the activity of an AML-selective *MYC* super enhancer by dismissing essential transcriptional coactivators

**Figure 2:**
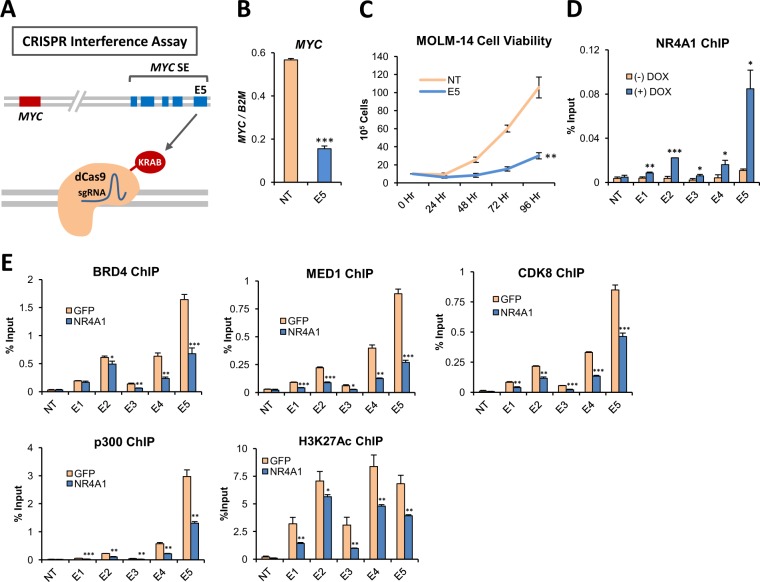
NR4A1 Disrupts the Activity of an AML-Selective *MYC* Super Enhancer by Dismissing Essential Transcriptional Coactivators. (**A**) Schematic illustrating CRISPR interference strategy involving the use of a guide RNA specific to the E5 enhancer, which targets dCas9-KRAB to disrupt the activation status of the enhancer. (**B**) RT-qPCR for *MYC* in MOLM-14 cells transduced with dCas9-KRAB lentivirus containing either a non-targeting (NT) sgRNA cassette, or sgRNA targeting the E5 enhancer. (**C**) Cell viability measured in MOLM-14 cells transduced with dCas9-KRAB lentivirus targeting NT or E5. (**D**) ChIP-qPCR for NR4A1 in MOLM-14 with dox-inducible lentiviral NR4A1 expression showing its enrichment across the *MYC* SE. NT refers to the negative control from a non-transcribed desert region. (**E**) ChIP-qPCR for BRD4, MED1, CDK8, p300 and H3K27Ac at 3 hours after electroporation with GFP or NR4A1 IVT RNA. Primer pairs were specific for the *MYC* SE E1-E5, and NT represents a non-transcribed gene desert negative control. ***p < 0.001, **p < 0.01, *p < 0.05 compared to GFP or vehicle controls.

To examine the mechanisms underlying NR4A dependent suppression of oncogene associated super enhancers, we focused on *MYC* since MLL-rearranged AMLs including MOLM-14 cells are highly dependent on *MYC* for leukemic maintenance. Using MLL-AF9 transduced murine bone marrow cells, previous studies have demonstrated that the AML-selective SE located 1.7 Mbp downstream from the *MYC* locus is required for AML maintenance in mice and is thought to drive *MYC* overexpression through long distance enhancer promoter looping interactions with the *MYC* promoter^5,19^. The SE is conserved in MLL-rearranged human AML cells including MOLM-14 and MV4–11 cells and consists of five unique enhancer elements, E1-E5 (Fig. 1J and Supplemental Fig. 6)^28^. Recent studies have shown that binding of hematopoietic master transcription factors across this genomic region activates the chromatin landscape through the recruitment of p300 histone acetyltransferase^20^. Subsequent enrichment for active histone mark H3K27Ac, as well as transcriptional coactivators BRD4 and Mediator (Supplemental Fig. 6) are thought to facilitate SE interactions with the *MYC* promoter to drive transcription elongation of *MYC*^20^. To determine whether the conserved *MYC* SE is also required for maintenance of *MYC* expression in human AML cells, we used a CRISPR-interference assay to target dCAS9-KRAB to the E5 enhancer in MOLM-14 cells^35^. We found that targeting of the E5 enhancer substantially reduced *MYC* transcription and led to diminished AML cell viability (Fig. 2A–C). Thus, the functional requirement for the MYC SE is conserved between mouse and human AML cells.

Forced expression of NR4A1 in MLL-rearranged MOLM-14 and MV4–11 cells results in substantial repression of *MYC* expression (Supplemental Fig. 7) and our analysis of the NR4A cistrome in MOLM-14 cells revealed strong NR4A recruitment to the E2 and E5 enhancers of the *MYC* SE after treatment with DHE. In contrast, forced expression of NR4A1 had no impact on *MYC* expression in K562 chronic myeloid leukemia cells, which which express high levels of *MYC* but do not display SE activation at this genomic region (Supplemental Fig. 8A–C). Furthermore, the viability of K562 cells was similarly unresponsive to DHE (Supplemental Fig. 8D). These observations suggest that NR4A1-dependent *MYC* suppression is selective for cells in which *MYC* is driven by the SE.

Given the essential role of the SE in maintaining *MYC* expression, we next asked if NR4A1 directly interferes with SE activity. Using ChIP-qPCR for NR4A1 in MOLM-14 cells expressing a dox-inducible NR4A1 construct, we confirmed that NR4A1 is recruited to the the *MYC* SE E1-E5 region (Fig. 2D). The activity of the *MYC* SE is highly dependent on the recruitment of transcriptional coactivators BRD4 and Mediator, which are most highly enriched at the *MYC* SE in MLL-AF9 AML cells and are co-enriched with p300 and p300-dependent H3K27Ac (Supplemental Fig. 9). To determine how NR4A1 binding across the *MYC* SE affects its activation status, we performed ChIP-qPCR for BRD4, MED1, CDK8, and p300 with its active histone acetylation mark H3K27Ac (Fig. 2E). In the absence of NR4A1, we observed high levels of coactivator recruitment and H3K27Ac, consistent with an activated *MYC* SE. Recruitment of each of these coactivators, as well as H3K27Ac, was significantly suppressed by NR4A1.

### NR4A1 disrupts transcriptional activity and enhancer-promoter looping of the *MYC* SE

**Figure 3:**
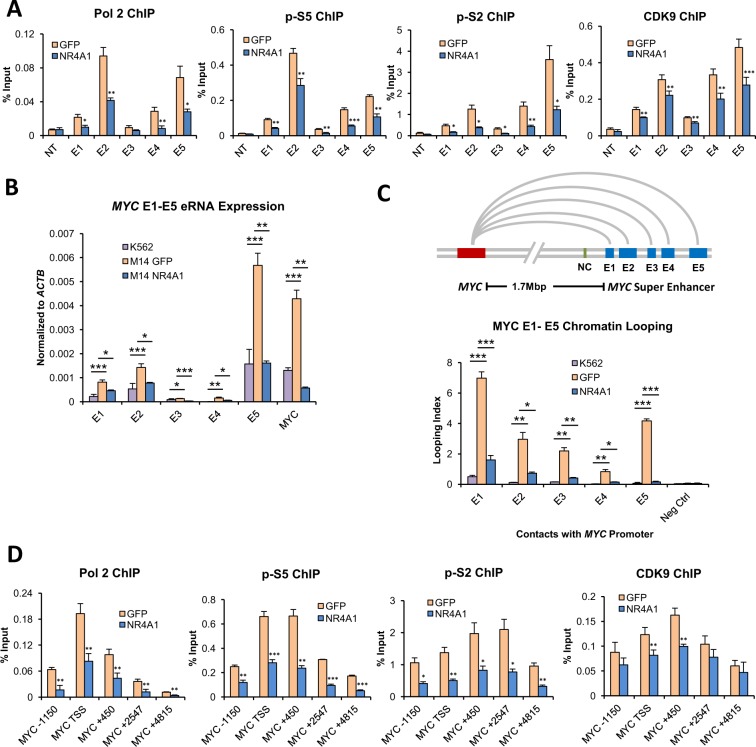
NR4A1 Disrupts Transcriptional Activity and Enhancer-Promoter Looping of the *MYC* SE. For (**A**–**C**) MOLM-14 cells were treated with vehicle or 10 uM DHE for 4 hours. (**A**) ChIP-qPCR for total RNA polymerase II (Pol 2), Pol 2 phospho serine 5 (p-S5), Pol 2 phospho serine 2 (p-S2), and CDK9 using primer pairs specific for the *MYC* SE E1-E5. (**B**) RT-qPCR for enhancer RNAs (eRNAs) from the *MYC* SE E1-E5 as well as *MYC* mRNA. K562 cells were included as a negative control as they do not express eRNA at E1-E5. (**C**) 3C chromatin looping demonstrating the frequency of interactions between the *MYC* SE E1-E5 and the *MYC* promoter, represented as looping index. K562 cells are included as a non-looping negative control. The green bar in the schematic labeled NC represents a genomic region between the *MYC* locus and the *MYC* SE which was used as an additional non-looping negative control. (**D**) ChIP-qPCR for total RNA polymerase II (Pol 2), Pol 2 phospho serine 5 (p-S5), Pol 2 phospho serine 2 (p-S2), and CDK9 at 3 hours after electroporation with GFP or NR4A1 IVT RNA. ChIP-qPCRs were done using primer pairs that span the *MYC* locus. ***p < 0.001, **p < 0.01, *p < 0.05 compared to vehicle, GFP or K562 controls.

Enhancers possess transcriptional activity and recruit RNA Pol 2 to promote transcription of non-coding enhancer RNAs (eRNAs)^36,37^. The recruitment of Pol 2 and phosphorylation of serine 5 of its c-terminal domain (CTD) initiates abortive transcription, generating only short incomplete RNA fragments^38,39^. Release of paused Pol 2 and activation of transcription elongation is mediated by the super elongation complex (SEC), including its functional module p-TEFb and its CDK9 kinase subunit^40,41^. The SEC phosphorylates the CTD of Pol 2 at serine 2, activating transcription elongation^39^. Recent studies have revealed that eRNAs are required for the expression of enhancer target genes^42,43^, including *MYC*^17^, and they promote recruitment of the super elongation complex (SEC)^44^, and enhancer-promoter looping^45^. To determine the effect of NR4A1 on *MYC* SE transcriptional activity, we performed ChIP-qPCR for total Pol 2, phospho serine 5 (p-S5), phospho serine 2 (p-S2) and CDK9 across the E1-E5 enhancers (Fig. 3A). We also measured E1-E5 eRNA expression using RT-qPCR (Fig. 3B). We observed a significant NR4A1-dependent reduction of active Pol 2 levels and CDK9, and consistent with these findings, we observed a marked reduction of eRNA expression. The reduced eRNA levels also mirror the suppression of *MYC* mRNA expression by NR4A1 (Fig. 3B). This observation is consistent with a model where transcription at cognate enhancer-promoter pairs is mutually coordinated^46^.

Regulation of target gene expression by distal enhancers is thought to be mediated through long range enhancer-promoter looping interactions^47^. Direct looping between the *MYC* promoter and the *MYC* SE has been demonstrated previously in mouse AML cells^5^. Using 3 C, we measured the frequency of looping between the *MYC* promoter and the SE E1-E5, which was significantly reduced by NR4A1 (Fig. 3C). Finally, loss of *MYC* SE activity in response to NR4A1 expression was accompanied by a loss of transcriptional activity at the *MYC* promoter as measured by ChIP-qPCR for total Pol 2, p-S5, p-S2, and CDK9 using primers spanning the *MYC* genomic locus (Fig. 3D). Thus, NR4A1 suppresses *MYC* transcription via direct interaction with the *MYC* SE where it dismisses essential coactivators, leading to significant reduction of SE transcriptional activity and enhancer-promoter looping.

### DHE and JQ1 suppress transcription progression at the *MYC* locus

**Figure 4:**
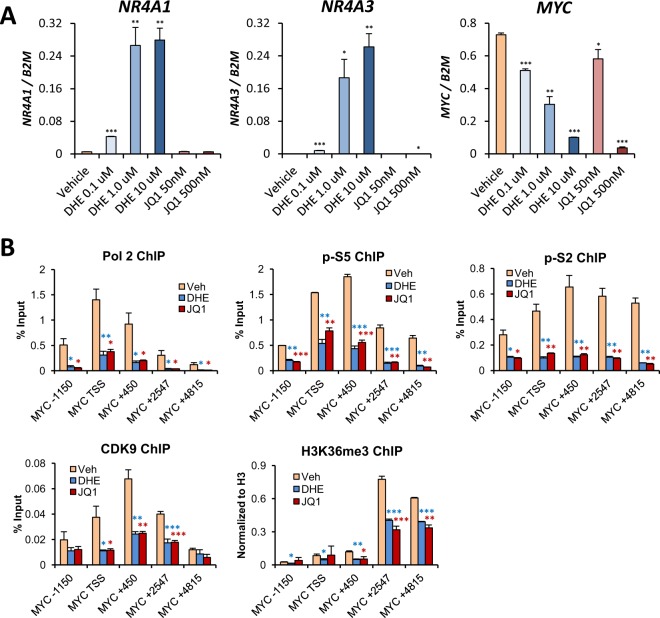
DHE and JQ1 suppress transcription progression across the *MYC* locus. (**A**) Dose responsive expression of *NR4A1*, *NR4A3* and *MYC* mRNAs using RT-qPCR in MOLM-14 cells treated with specified concentrations of DHE or JQ1 for 6 hours. (**B**) ChIP-qPCR for total RNA polymerase II (Pol 2), Pol 2 phospho serine 5 (p-S5), Pol 2 phospho serine 2 (p-S2), CDK9, and H3K36me3. ChIP-qPCRs were done using primer pairs that span the *MYC* locus at 4 hours after treatment with 10 uM DHE or 500 nM JQ1. ***p < 0.001, **p < 0.01, *p < 0.05 compared to vehicle controls.

To further understand the mechanisms underlying SE suppression by DHE, we focused on the *MYC* SE. We compared the activity of DHE to that of the BET inhibitor JQ1, which is highly efficacious at repressing *MYC* overexpression in AML^20,29,48,49^ and suppresses *MYC* SE activation via dismissal of BRD4 and Mediator^30^. This approach allows for a side-by-side comparison between the two drugs to compare their molecular mechanisms and pre-clinical efficacies. Analysis of the transcriptional responses of NR4As to DHE and JQ1 demonstrated that while both drugs similarly suppress *MYC* transcription in a dose-dependent manner, JQ1 has no effect on *NR4A* expression, thereby eliminating NR4As as contributors to JQ1-dependent *MYC* suppression (Fig. 4A).

We next compared the effects of DHE and JQ1 on transcription at the *MYC* locus. Consistent with our previous results with NR4A1, we observed robust decreases in pro-transcriptional markers at the *MYC* locus, including Pol 2, p-S5, p-S2 and CDK9 with either DHE or JQ1 treatment (Fig. 4B). This loss of transcriptional progression correlated with a suppression of intragenic H3K36me3 recruitment, an epigenetic marker of transcription elongation^50^.

### DHE mimics NR4A1-dependent suppression of the *MYC* SE and displays similar efficacy to bet inhibitor JQ1

**Figure 5:**
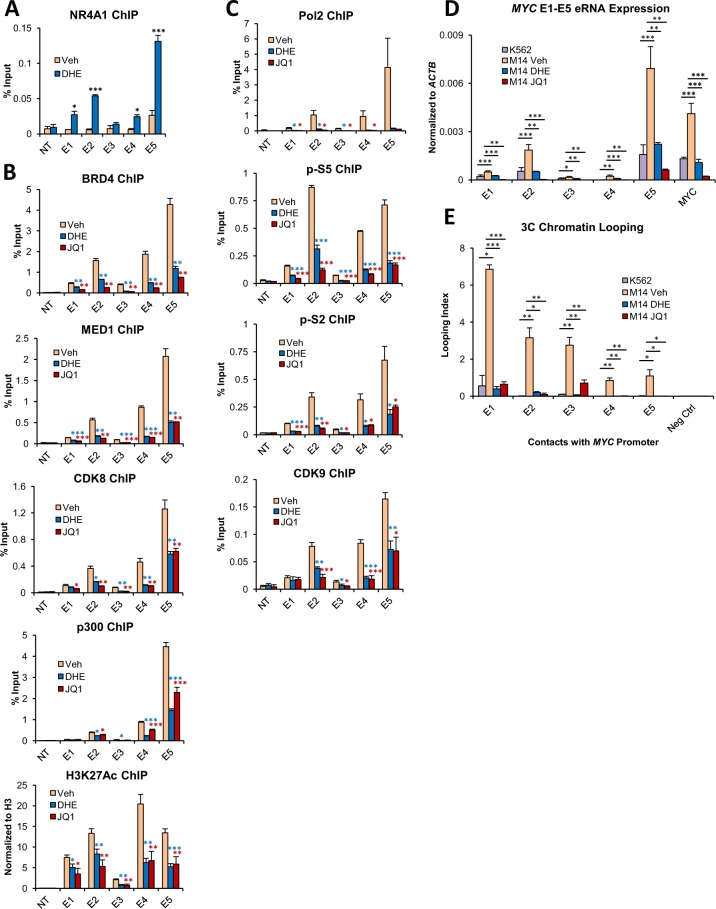
DHE mimics NR4A-dependent suppression of the *MYC* SE and displays similar efficacy to BET inhibitor JQ1. MOLM-14 cells were treated with 10 uM DHE or 500 nM JQ1 for 4 hours. (**A**) ChIP-qPCR for NR4A occupancy at the *MYC* SE in response to DHE treatment. (**B**) ChIP-qPCR for BRD4, MED1, CDK8, p300 and H3K27Ac. (**C**) ChIP-qPCR for total RNA polymerase II (Pol 2), Pol 2 phospho serine 5 (p-S5), Pol 2 phospho serine 2 (p-S2), and CDK9. ChIP-qPCRs for (**A**–**C**) were done using primer pairs specific for the *MYC* SE E1-E5 enhancers. NT represents a non-transcribed gene desert negative control. (**D**) RT-qPCR for enhancer RNAs (eRNAs) from the *MYC* SE E1-E5 as well as *MYC* mRNA in MOLM-14 cells. K562 cells are included as a negative control for eRNA expression at the *MYC* SE. (**E**) 3C chromatin looping frequency of interactions between the *MYC* SE E1-E5 and the *MYC* promoter. K562 cells are included as a non-looping negative control. Also included is a non-looping negative control genomic region between the *MYC* locus and the *MYC* SE. ***p < 0.001, **p < 0.01, *p < 0.05 compared to vehicle or K562 controls.

The dismissal of MED1 from the *MYC* SE by DHE suggested that DHE might recapitulate NR4A1-dependent suppression of the *MYC* SE. We first confirmed using ChIP-qPCR that DHE stimulates the enrichment of NR4As at the *MYC* SE (Fig. 5A). Next, we examined the effects of both drugs on recruitment of SE coactivators required for maintenance of SE activation. Treatment with either DHE or JQ1 significantly reduced the enrichment of coactivators BRD4, MED1, CDK8 and p300, in addition to the active enhancer mark H3K27Ac (Fig. 5B). We noted that while JQ1 is a direct inhibitor of BRD4, enrichment of MED1 and additional coactivators was also reduced, which is consistent with previous studies^13,30^. Treatment with either drug had no significant impact on the transcriptional levels of these coactivators (Supplemental Fig. 10A). Additionally, DHE disruption of coactivator enrichment across the *MYC* SE is dose-dependent (Supplemental Fig. 10B). We also confirmed that MV4–11 cells respond similarly to MOLM-14, with significant reduction in coactivator recruitment at the *MYC* SE following DHE or JQ1 exposure (Supplemental Fig. 11). Disruption of coactivator enrichment at the *MYC* SE in response to drug exposure was accompanied by significant loss of E1-E5 transcriptional activity, including significant reductions of active Pol 2 levels, CDK9 and eRNA expression (Fig. 5C,D). Finally, treatment with DHE or JQ1 substantially reduced looping between the *MYC* promoter and the E1-E5 enhancers (Fig. 5E). From these results we concluded that DHE and JQ1 comparably suppress the activation status of the *MYC* SE.

### DHE and JQ1 suppress tumor growth and intratumoral *MYC* expression in a xenograft mouse model of MLL-rearranged AML

**Figure 6:**
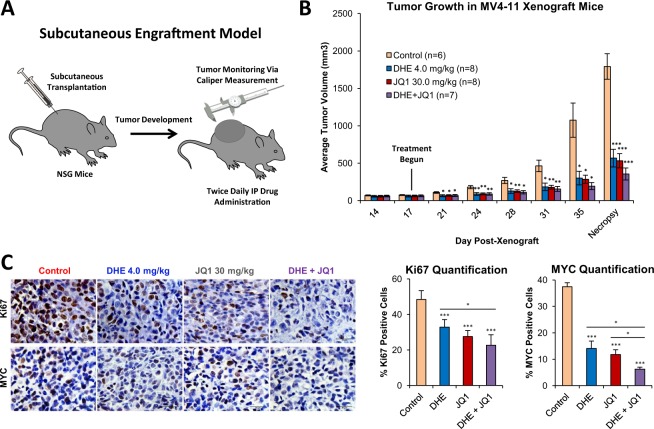
DHE and JQ1 suppress tumor growth and intratumoral *MYC* expression in a xenograft mouse model of MLL-rearranged AML. (**A**) Schematic depicting subcutaneous xenograft strategy. (**B**) 7 week-old NSG mice were transplanted with 1 × 10^7^ MV4–11 cells, and monitored until average tumor volume reached 60 mm^3^, at which point intraperitoneal injections of vehicle, 4.0 mg/kg DHE, 30 mg/kg JQ1, or the two drugs combined were administered twice daily. Average tumor volume is indicated. (**C**) Immunohistochemical staining and quantification of proliferative marker Ki67, and *MYC* in MV4–11 xenograft tumor tissues. ***p < 0.001, **p < 0.01, *p < 0.05 compared to vehicle controls.

We have previously shown that DHE delays AML progression and promotes myeloid differentiation in a dose-dependent manner in a xenograft mouse model of disseminated MLL- rearranged human AML^33^. To determine whether the antileukemic effects of DHE were associated with suppression of expression of intratumoral *MYC in vivo*, we implemented an alternative xenograft mouse model using subcutaneous engraftment of human AML cells, which is a validated approach for monitoring intratumoral biomarkers of drug response, including suppression of *MYC*^51^. MV4–11 AML cells were transplanted subcutaneously into NOD/SCID/gamma (NSG) mice and tumors were allowed to develop to 60 mm^3^ in size. To compare the effects of DHE and JQ1 on tumor growth and *MYC* expression, mice were then treated twice daily via intraperitoneal injections with vehicle control (DMSO), DHE (4 mg/kg), JQ1 (30 mg/kg), or DHE and JQ1 combined, and tumor size was monitored using calipers. When the first animal within a treatment group reached the institutional limits for tumor size, all animals were sacrificed and tumors were excised for analysis (Fig. 6A). Treatment with either drug resulted in similar *in-vivo* tumor growth suppression, which was approximately 70% reduced at necropsy (Fig. 6B). We also observed comparable reduction of Ki67 staining in both DHE and JQ1 treatment groups. Tumors with either DHE or JQ1 showed roughly 60% reduction in intratumoral *MYC* staining, and around 75% reduction when the two drugs were combined (Fig. 6C). While we did not observe a substantial additive effect on tumor growth inhibition when DHE and JQ1 were combined, this supports the idea that both drugs converge on the same molecular target, namely the *MYC* SE. Finally, the tumor growth suppressive effects of DHE also extended to a MOLM-14 cell xenograft model of MLL-rearranged AML and were DHE dose-responsive at clinically-relevant doses of DHE (Supplemental Fig. 12).

## Discussion

Enhancer reprogramming in AML leads to the acquisition of super enhancers (SE) that drive the expression of oncogenes, contributing to AML progression and chemotherapeutic resistance. These SEs represent promising targets for therapeutic intervention. While recent studies have disclosed the identity of master transcription factors and chromatin modifiers required for SE maintenance, the identity of mediators and mechanisms of SE repression are less well understood. We have identified a novel DHE-activated NR4A nuclear receptor signaling axis that represses super enhancer-dependent oncogene expression in AML cells. Both NR4A1 and the NR4A activating drug, DHE, regulate overlapping gene expression programs in MLL-AF9 rearranged human AML cells, and repress the expression of a select group of SE-associated leukemic oncogenes including *MYC*. Focusing on the *MYC* SE as a model to understand the mechanisms underlying NR4A1 repression of SE-dependent oncogene expression, we demonstrated that 1) the *MYC* SE is functionally required for maintenance of *MYC* expression and to sustain leukemic growth of MLL-AF9 rearranged human AML cells, 2) NR4A1 is recruited to the constituent enhancer elements of the SE and represses *MYC* transcription by suppressing essential coactivator recruitment to the SE, leading to disruption of enhancer-promoter looping and promoter stalling of Pol 2, 3) DHE induction of endogenous NR4A expression recapitulates NR4A1-dependent repression of *MYC* by decommissioning the activation status of the *MYC* SE and 4) DHE shows similar efficacy compared to the BET bromodomain inhibitor JQ1 at repressing SE-dependent expression of *MYC in vitro* and suppressing growth of *MYC*-dependent MLL-rearranged human AML *in vivo* in a mouse xenograft model.

Recent whole genome occupancy analysis of BRD4, Mediator and p300 in MLL-AF9 AML cells revealed that these factors co-occupy active H3K27 acetylated enhancers and exhibit asymmetric high level loading at SEs^20,28,30^. SE activation is highly sensitive to interference in occupancy of these coactivators^13^ and knockdown or pharmacological inhibition of p300, MED1 or BRD4 decommissions the activation status of SEs and leads to repression of associated target genes, including oncogenic drivers of AML cells^20,28–31,52^. We used MED1 as a surrogate to distinguish SEs from typical enhancers and monitor their sensitivity to DHE^11,12,30^. We identified 290 SEs in AML cells and, using ChIP-Seq analysis for NR4A occupancy, we found that DHE exposure led to recruitment of endogenous NR4As to nearly all SEs identified. Further, DHE reprogrammed MED1 occupancy at a select subset of NR4A1-bound SEs and dismissed MED1 binding from a select group of SE-associated key leukemic oncogenic drivers whose transcription is repressed by both NR4A1 and DHE. Notably, the most significantly repressed SE-associated oncogenes identified in this study, including *MYC*, *BCL2* and *CDK6*, were previously shown to be highly sensitive to BET inhibitors^49^. Further, transcriptional repression by BET inhibitor JQ1 was highly correlated with the degree of MED1 loss from a subset of MED1-associated SEs^30^. These findings reveal that overlapping mechanisms underlie repression SE-dependent gene expression at the level of MED1 suppression by both BRD4 inhibition and NR4A activation.

Given the central role of *MYC* as a key oncogenic driver of AML, and the identification of a conserved SE predicted to control *MYC* expression in a broad range of cytogenetically distinct AMLs, we focused on this gene to understand how NR4A1 and DHE regulate SE-dependent *MYC* expression. Activation of the *MYC* SE is conserved between mouse and human AMLs^5,20,28,53^. Functional requirement for the SE in maintenance of MLL-AF9 driven murine AML has recently been demonstrated^19^ and we confirmed using CRISPR/Cas9 interference of the SE that this requirement extends to human MLL-AF9-driven AML. Upon forced expression of NR4A1 in human AML cells, we found that binding of NR4A1 at the *MYC* SE is sufficient to decommission the activation status of the SE by suppressing the recruitment of essential coactivators including BRD4, Mediator and p300, leading to loss of p300-dependent H3K27 acetylation and Pol 2-dependent eRNA transcription. NR4A suppression of SE activation also leads to loss of enhancer-promoter looping and recruitment of coactivators required for transcription progression at the *MYC* locus. Recruitment of coactivators to the *MYC* SE is dictated by enhancer-bound master hematopoietic transcription factors that recruit p300 to provide an acetylated platform for binding of BRD4 to maintain activation of the SE. Further, inhibition of p300 causes loss of H3K27 acetylation and suppression of BRD4 occupancy, leading to SE inactivation^20^. The NR4A1-dependent dismissal of p300, and reduction of H3K27Ac at the SE, therefore provides a mechanism underlying loss of BRD4 occupancy and SE deactivation. However, the precise mechanism by which NR4A1 recruitment promotes dismissal of p300 remains to be established.

As predicted by the comparative ability of DHE and JQ1 to dismiss MED1 and repress transcription of select SE-associated oncogenes including *MYC*, we found that DHE and JQ1 showed comparable efficacy in repressing SE-dependent expression of *MYC in vitro*, and suppressing growth of *MYC*-dependent MLL-rearranged human AML *in vivo* in a mouse xenograft model. DHE-dependent induction of endogenous NR4A expression closely recapitulated the SE-dependent mechanism of NR4A-driven *MYC* repression. Further, while JQ1 is a selective inhibitor of BET proteins^54^, it affected not only the enrichment of BRD4 at the *MYC* SE, but also dismissed Mediator and p300. This is consistent with previous studies showing that BET inhibition disrupts the binding of Mediator^30^, in addition to reducing the levels of active enhancer mark H3K27Ac at the *MYC* SE^53^. The comparable efficacy of DHE and JQ1 in mouse xenografts of human AML, and their overlapping molecular mechanisms of *MYC* suppression, predict that DHE may provide a viable alternative to BET inhibitors in select AML patients. While BET inhibitors function as suppressors of global super enhancer activity^13^, DHE selectively induces transcription of NR4A nuclear receptors^33^, which in turn bind to and suppress the activation status of a subset of AML SEs including the AML-selective *MYC* SE. Early clinical trials for BET inhibitors have thus far yielded mixed results^55^, and long-term BET inhibition has been associated with multiple toxicities in animal models^56^, in addition to BET inhibitor-induced drug resistance^53,57^. In contrast, DHE is a well-tolerated FDA approved drug and, as we highlighted in our recent study, long-term DHE administration has no negative impact on hematopoietic stem cell growth and differentiation in mice^33^. Combined, our results suggest that DHE administration may be a favorable alternative to BET inhibitors in AML patients with high super enhancer-driven *MYC* expression.

Finally, the *MYC* repressive effects of NR4A1 and DHE we observed in this study were cell-selective and not observed in leukemic cells lacking the *MYC* SE^53^. These findings, if substantiated in AML patient cohorts, suggest that epigenetic stratification of AML patients based on identification of a functional *MYC* SE, as measured by eRNA expression, may provide a viable approach to predict patient response to NR4A-directed therapy.

## Supplementary information

Supplementary information.

Supplementary information 2.
