# Supplementary material for: Targeting Oncogenic Super Enhancers in MYC-Dependent AML Using a Small Molecule Activator of NR4A Nuclear Receptors
Source: Sci Rep. 2020 Feb 18;10:2851. doi: 10.1038/s41598-020-59469-3 (PMC7029036; doi:10.1038/s41598-020-59469-3)
Supplement: Supplementary file 1 — Supplementary information. [file 41598_2020_59469_MOESM1_ESM.pdf]

Title: Targeting Oncogenic Super Enhancers in *MYC*-Dependent AML Using a Small Molecule Activator of NR4A Nuclear Receptors

Authors: Steven G. Call,[1-2] Ryan P. Duren,[1,3] Anil K. Panigrahi,[1] Loc Nguyen,[1] Pablo R. Freire,[1-2] Sandra L. Grimm,[1,4,5] Cristian Coarfa,[1,4,5] Orla M. Conneely[1,4,*]

[1]Department of Molecular and Cellular Biology, Baylor College of Medicine, Houston, TX 77030, USA

[2]Molecular and Cellular Biology PhD Program, Baylor College of Medicine, Houston, TX 77030, USA

[3]Integrative Molecular and Biomedical Sciences PhD Program, Baylor College of Medicine, Houston, TX 77030, USA

[4]Dan L Duncan Comprehensive Cancer Center, Baylor College of Medicine, Houston, TX 77030, USA

[5]Center for Precision Environmental Health, Baylor College of Medicine, Houston, TX 77030, USA

*Corresponding Author:

Orla Conneely, PhD

BCM-Michael Debakey Center, Room M511, MS: BCM130

Phone: 713-798-6233

Email: orlac@bcm.edu

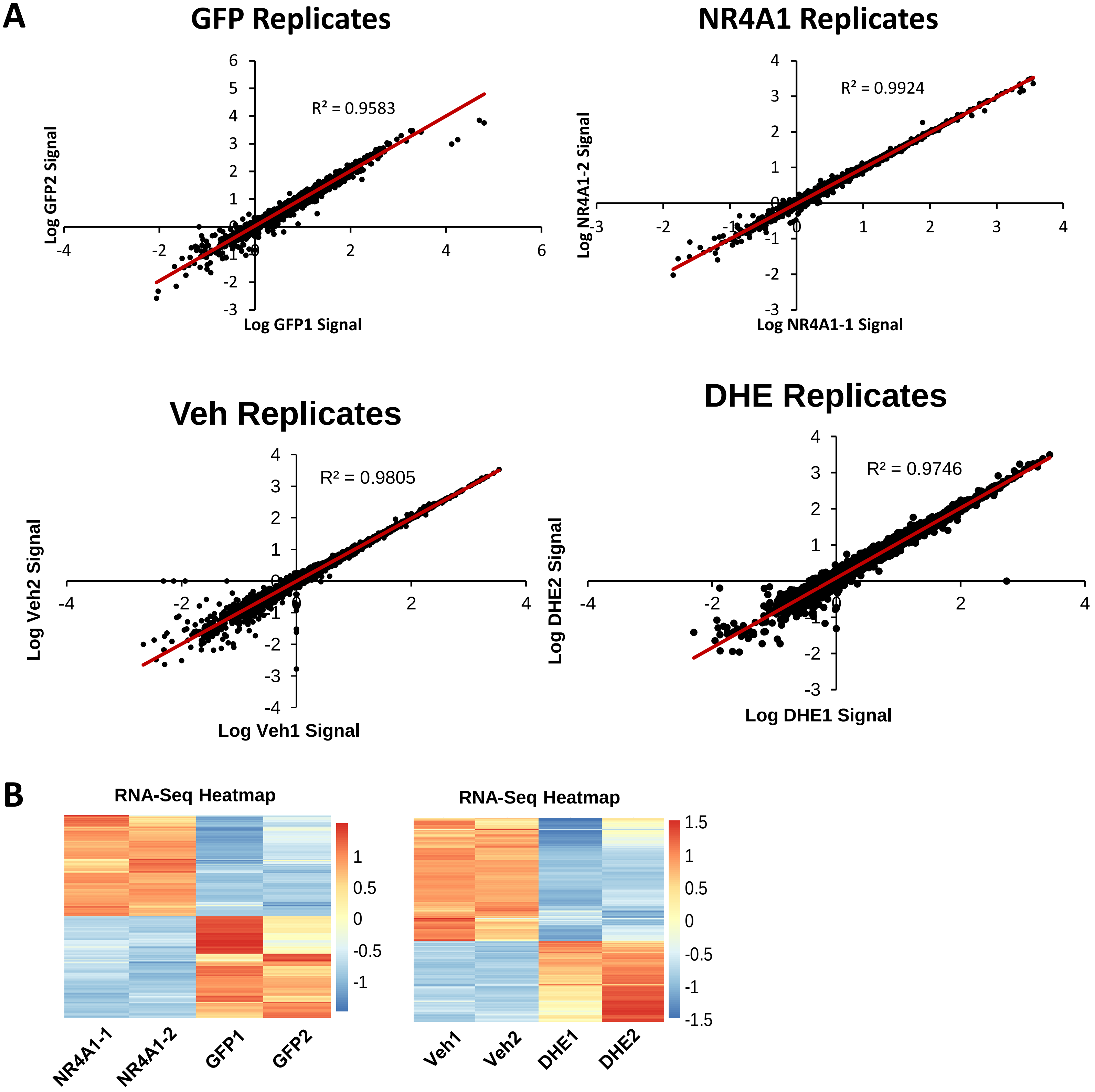

Supplemental Figure 1: RNA-Seq in MOLM-14 Cells with NR4A1 or DHE Treatment. For analysis of acute changes in gene expression in response to NR4A1 or DHE, MOLM-14 cells were electroporated with GFP control or NR4A1 IVT RNA, or treated with vehicle or 10uM DHE, and incubated for 6 hours. (A) Correlation between RNA-Seq treatment replicates (GFP/NR4A1, Veh/DHE) calculated using R^2. (B) Heatmap summary of RNA-Seq data, highlighting top 500 upregulated and downregulated genes at 6 hours following electroporation with GFP/NR4A1, or treatment with Veh/DHE.

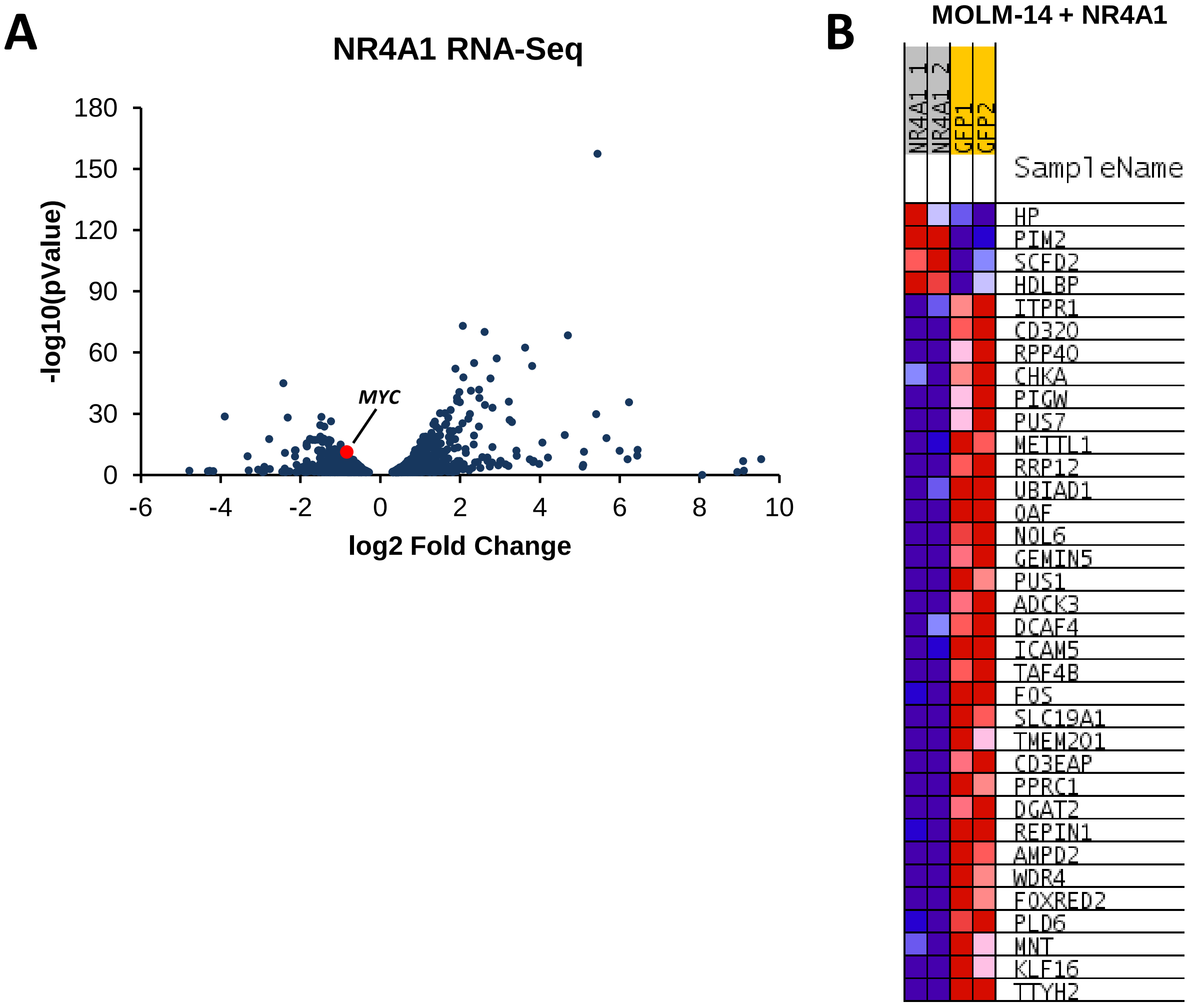

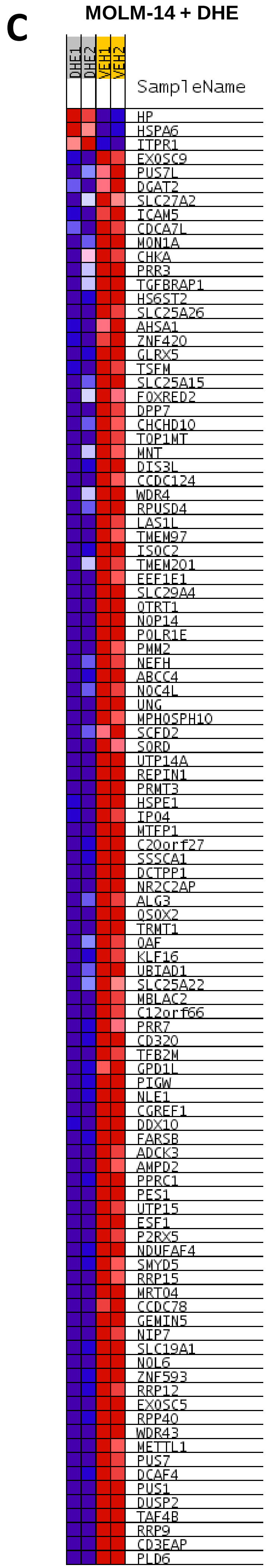

Supplemental Figure 2: NR4A1 and DHE similarly suppress *MYC* pathway genes. (A) Volcano plot summary of RNA-Seq results in MOLM-14 with GFP and NR4A1 IVT RNA. The red dot highlights *MYC* as a significantly repressed target of NR4A1. (B-C) RNA-Seq heatmaps generated using GenePattern from the Broad Institute, highlighting genes within the *MYC* pathway affected by NR4A1 (B) or DHE treatment (C).

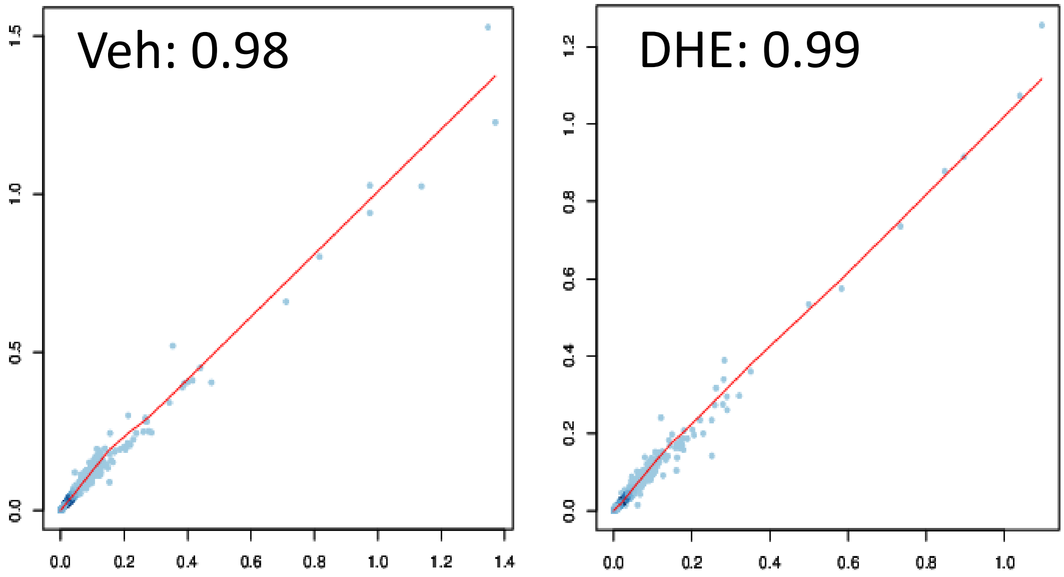

Supplemental Figure 3: Correlation of MED1 ChIP-Seq replicates. Pearson correlation of MED1 vehicle and DHE ChIP-Seq replicates.

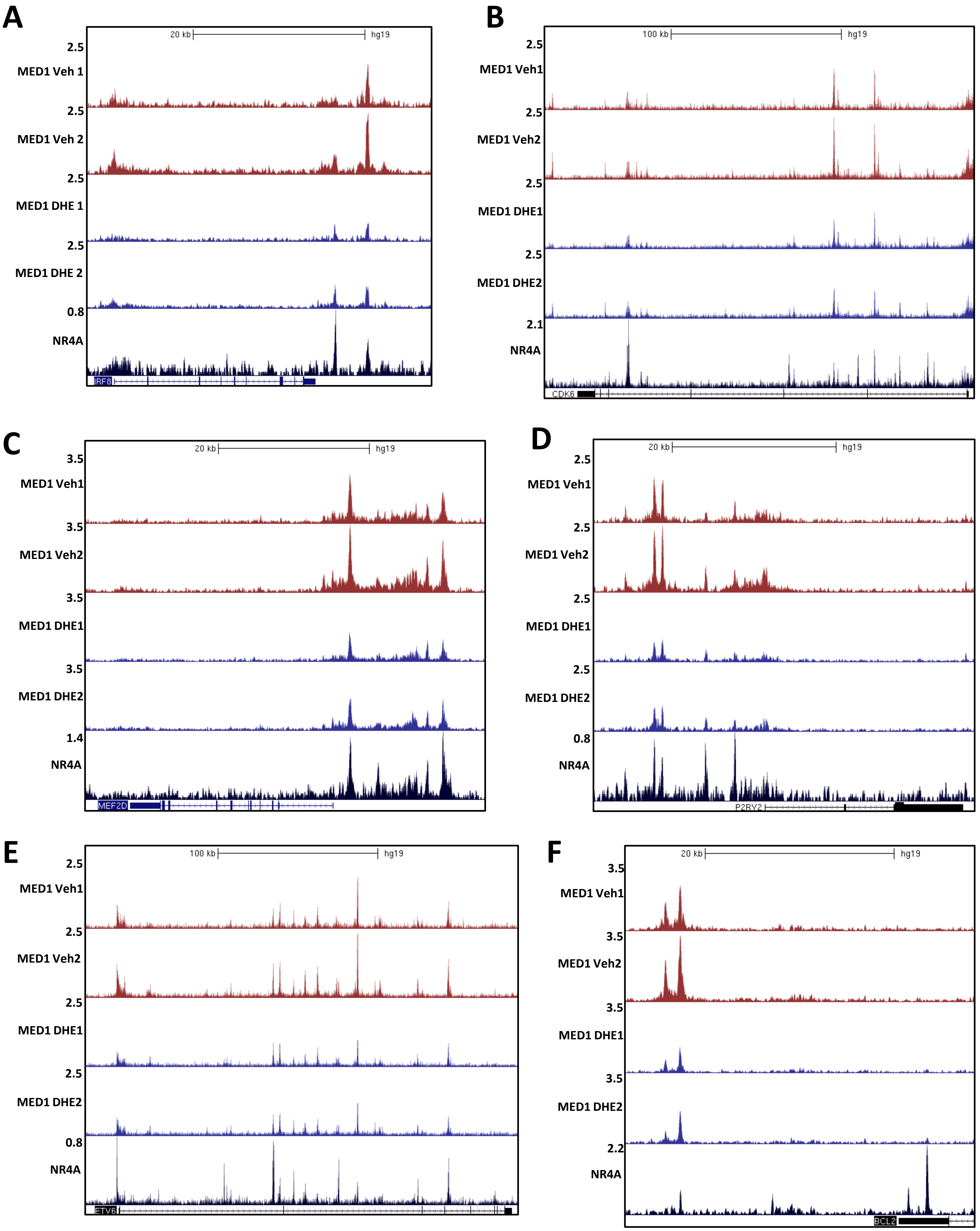
A
20 kb
hg19
2.5
MED1 Veh 1
2.5
MED1 Veh 2
2.5
MED1 DHE 1
2.5
MED1 DHE 2
0.8
NR4A
IRF8
B
100 kb
hg19
2.5
MED1 Veh1
2.5
MED1 Veh2
2.5
MED1 DHE1
2.5
MED1 DHE2
2.1
NR4A
CDK6
C
20 kb
hg19
3.5
MED1 Veh1
3.5
MED1 Veh2
3.5
MED1 DHE1
3.5
MED1 DHE2
1.4
NR4A
MEF2D
D
20 kb
hg19
2.5
MED1 Veh1
2.5
MED1 Veh2
2.5
MED1 DHE1
2.5
MED1 DHE2
0.8
NR4A
P2RY2
E
100 kb
hg19
2.5
MED1 Veh1
2.5
MED1 Veh2
2.5
MED1 DHE1
2.5
MED1 DHE2
0.8
NR4A
ETV6
F
20 kb
hg19
3.5
MED1 Veh1
3.5
MED1 Veh2
3.5
MED1 DHE1
3.5
MED1 DHE2
2.2
NR4A
BCL2

Supplemental Figure 4: DHE reduces MED1 signal across a subset of super enhancers in MOLM-14 cells. (A-F) UCSC Genome Browser ChIP-Seq screenshots for MED1 in MOLM-14 with Veh, or DHE treatment, in addition to ChIP-Seq for NR4A in MOLM-14 treated with DHE. These screenshots show loss of Mediator signal with DHE treatment at super enhancers regulating (A) *IRF8*, (B) *CDK6*, (C) *MEF2D*, (D) *P2RY2*, (E) *ETV6*, and (F) *BCL2*. Inclusion of NR4A ChIP-Seq highlights overlap of NR4A peaks with sites of MED1 loss.

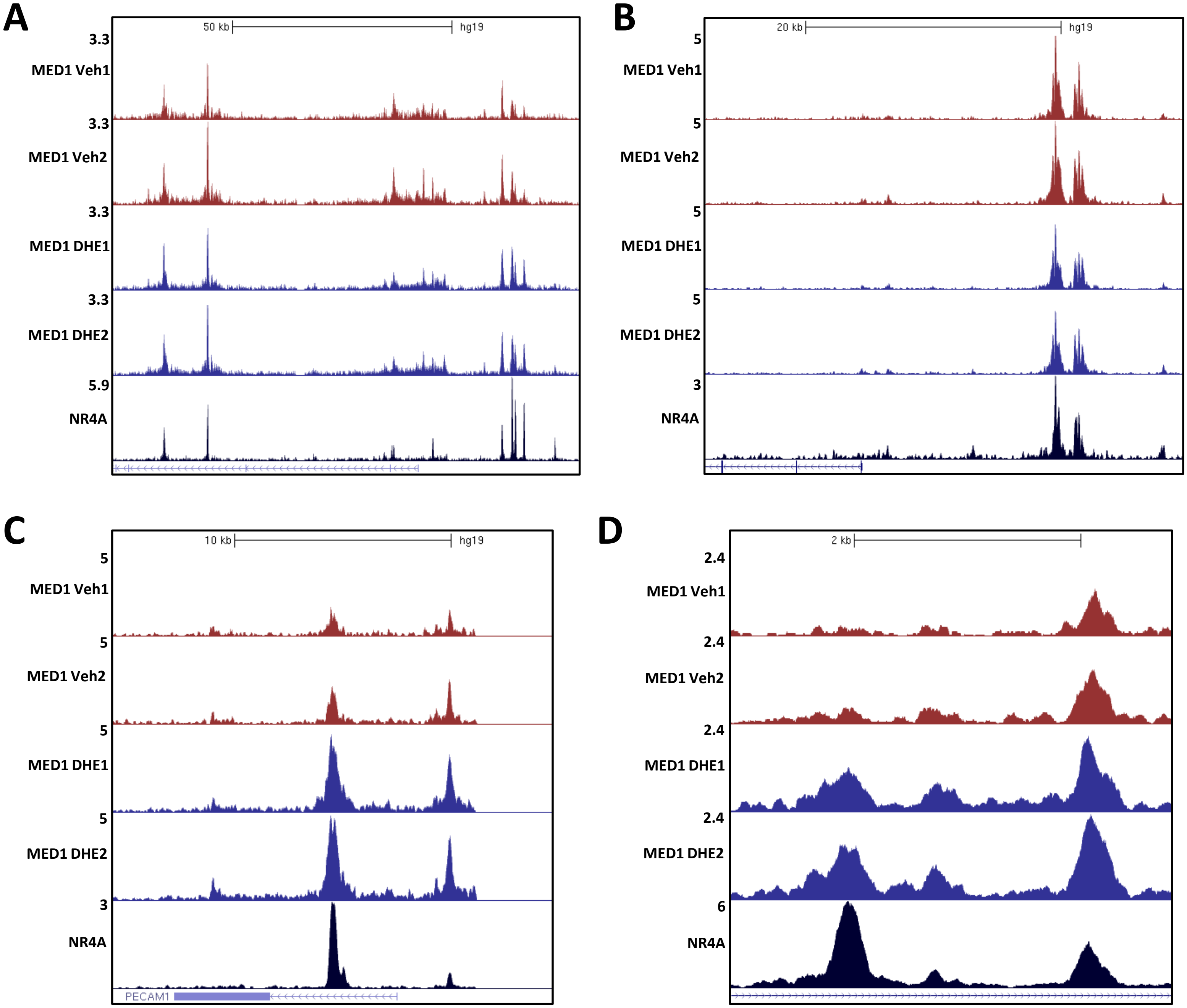

Supplemental Figure 5: NR4As are also associated with SEs that are unchanged or enhanced by DHE treatment. UCSC genome browser screenshots of super enhancers unchanged by DHE treatment include (A) *ATP8B4* and (B) *GRAP*. Screenshots of super enhancers that have increased signal in response to DHE treatment include (C) *PECAM1* and (D) *UBAC2*.

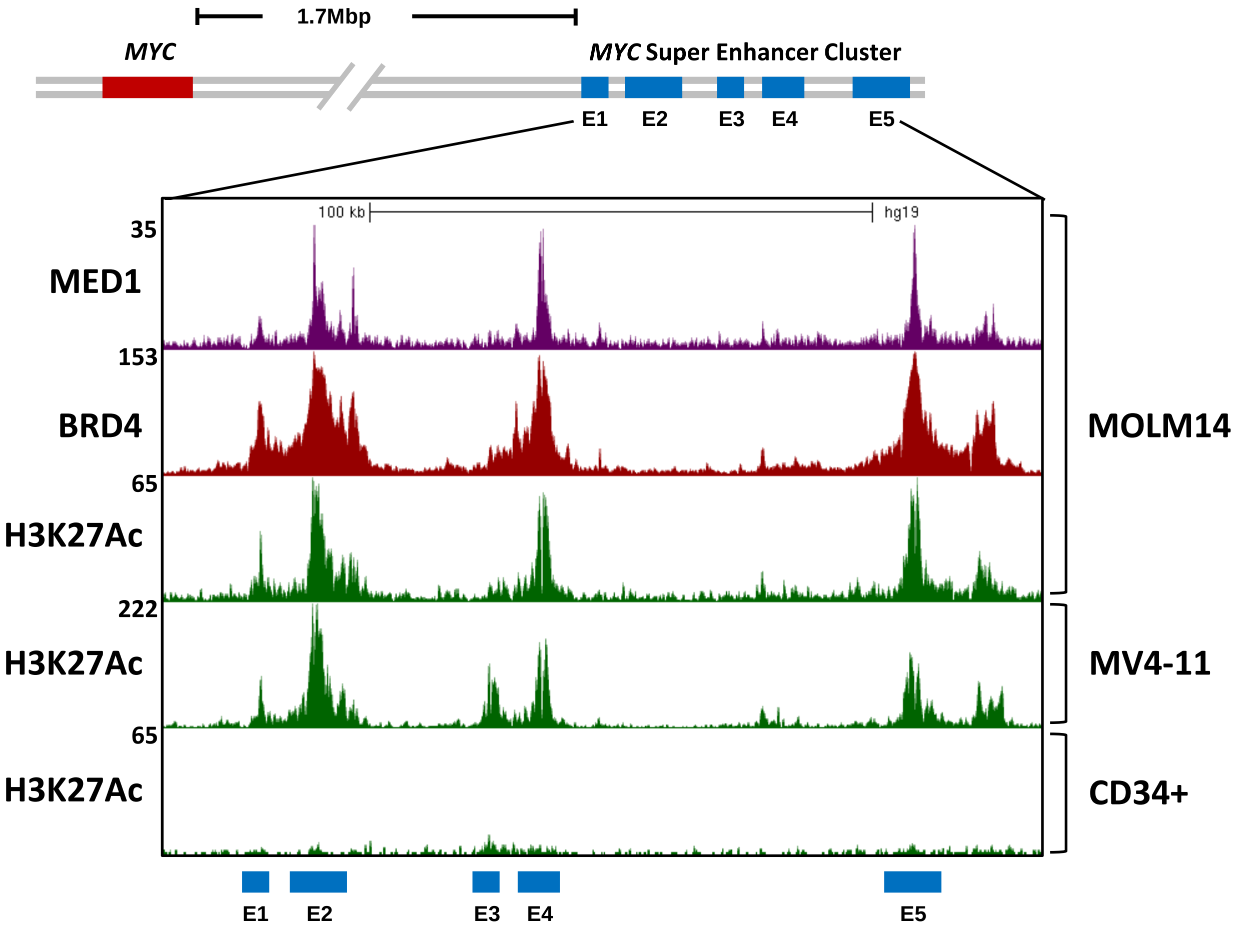

Supplemental Figure 6: An AML-selective *MYC* super enhancer is identified by its coactivator enrichment. (A) ChIP-seq for MED1, BRD4 and H3K27Ac in MOLM-14 human AML cells, compared to ChIP-Seq for H3K27Ac in MV4-11 cells and H3K27Ac in CD34+ human hematopoietic stem/progenitor cells, showing the activation status of the enhancer landscape is specific to AML.

ChIP-Seq for MED1, BRD4 and H3K27Ac in MOLM-14, and ChIP-Seq for H3K27Ac in MV4-11, were obtained from the following reference:

Pelish, H. E., Liau, B. B., Nitulescu, Ii, Tangpeerachaikul, A., Poss, Z. C., Da Silva, D. H., Caruso, B. T., Arefolov, A., Fadeyi, O., Christie, A. L., et al. 2015. Mediator kinase inhibition further activates super-enhancer-associated genes in AML. Nature, 526, 273-6.

ChIP-Seq for CD34+ H3K27Ac was obtained from the following reference:

Chacon, D., Beck, D., Perera, D., Wong, J. W. & Pimanda, J. E. 2014. BloodChIP: a database of comparative genome-wide transcription factor binding profiles in human blood cells. Nucleic acids research, 42, D172-7.

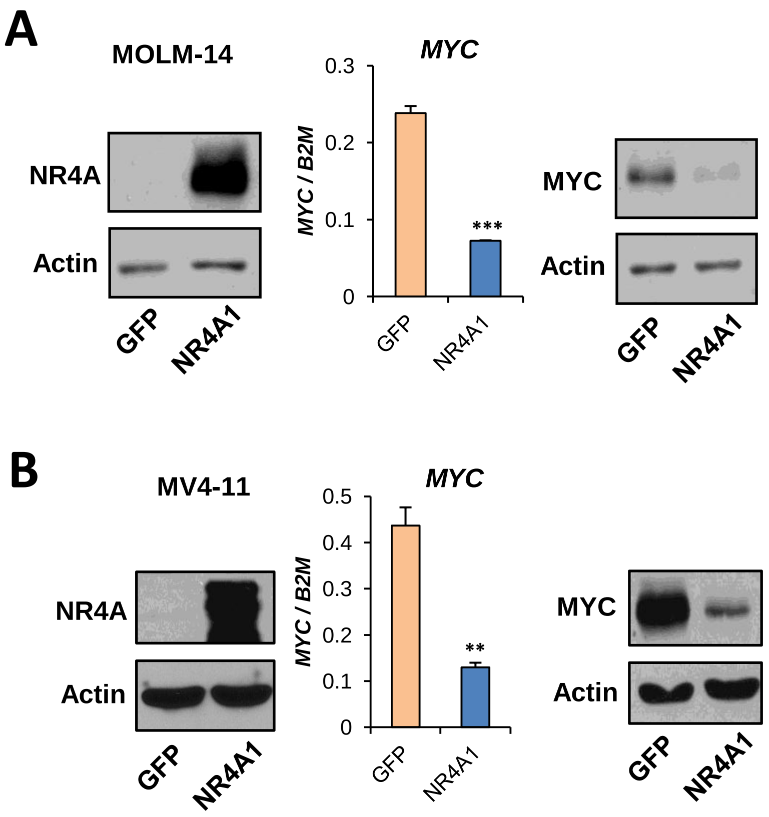

Supplemental Figure 7: NR4A1 suppresses *MYC* in MLL-rearranged human AML cell lines. RT-qPCR and Western blot analysis of NR4A1-dependent repression of *MYC* mRNA and protein in (A) MOLM-14 and (B) MV4-11 AML cells. ***p<0.001, **p<0.01 compared to GFP controls.

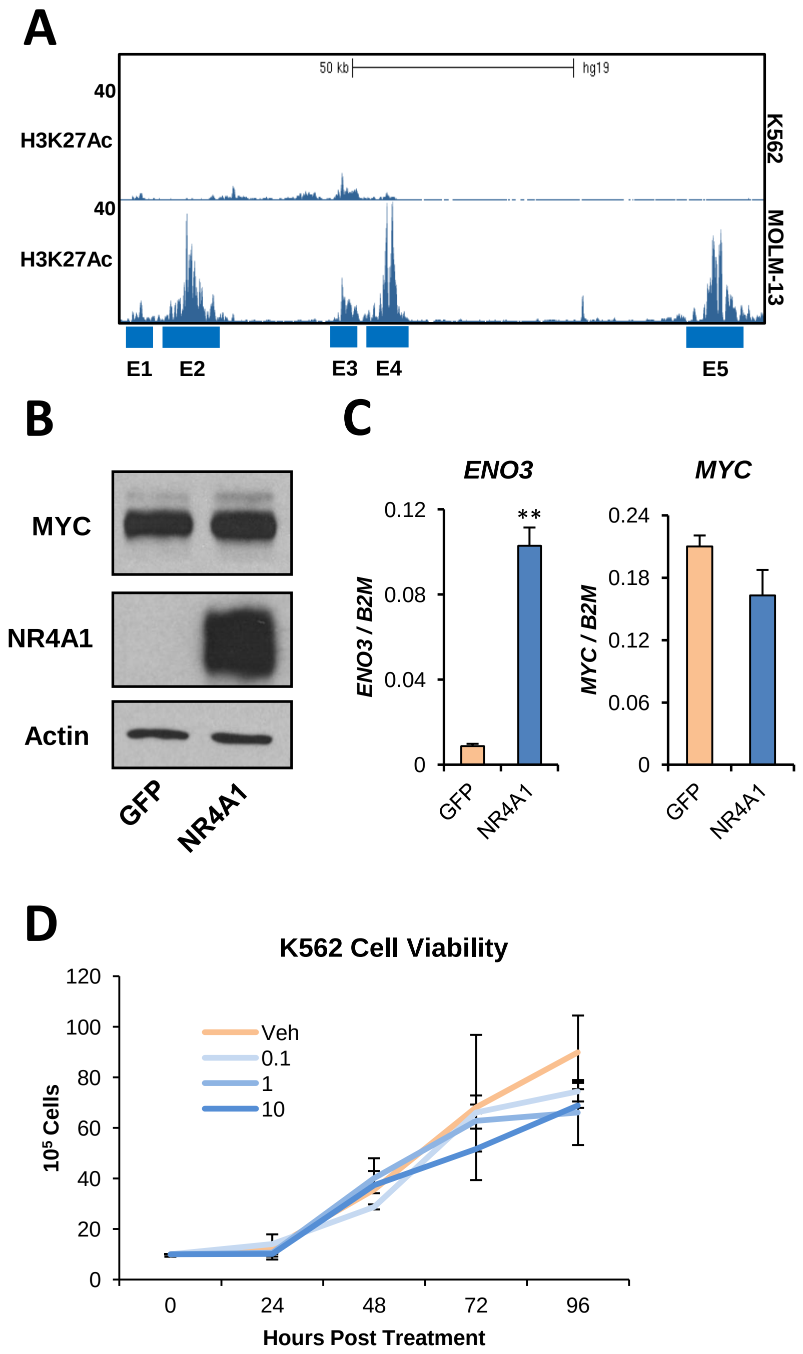

Supplemental Figure 8: K562 chronic myeloid leukemia cells lacking *MYC* SE activity are unresponsive to either NR4A1 expression or DHE treatment. (A) ChIP-Seq for H3K27Ac in K562 chronic myeloid leukemia (CML) cells compared to MOLM-13 AML cells, showing absence of active *MYC* SE landscape at this genomic region in K562. (B) MYC and NR4A1 protein levels in K562 cells with NR4A1 IVT RNA expression and measured using western blot. (C) RT-qPCR for *MYC* and *ENO3* transcript levels in K562 with IVT NR4A1. *ENO3* is a known upregulated target of NR4As in AML cells. (D) Cell viability measured in K562 treated with vehicle or DHE (0.1, 1.0, or 10 uM doses) for 96 hours. ***p*<0.01 compared to GFP controls.

ChIP-Seq for H3K27Ac in MOLM-13 and K562 was obtained from the following reference:

Rathert, P., Roth, M., Neumann, T., Muerdter, F., Roe, J. S., Muhar, M., Deswal, S., Cerny-Reiterer, S., Peter, B., Jude, J., et al. 2015. Transcriptional plasticity promotes primary and acquired resistance to BET inhibition. Nature, 525, 543-7.

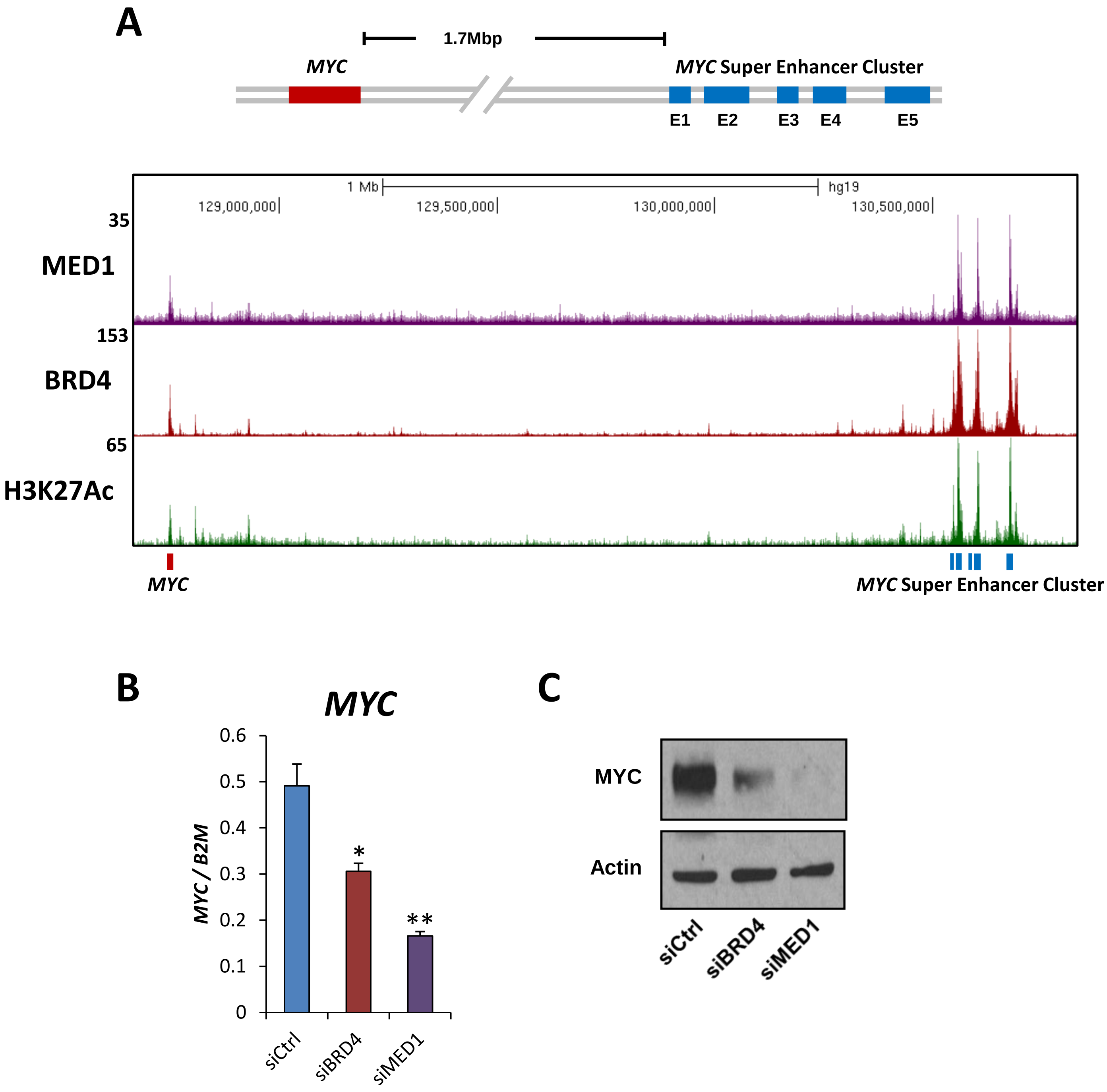

Supplemental Figure 9: BRD4 and Mediator are essential contributors to *MYC* maintenance. (A) UCSC Genome Browser screenshot for ChIP-Seq data in MOLM-14, including MED1, BRD4 and H3K27Ac, comparing enrichment at the *MYC* SE to the *MYC* promoter (Taken from Pelish, et al *Nature* 2015). *MYC* expression was measured in MOLM-14 using (B) RT-qPCR and (C) western blot in MOLM-14 cells 48 hours following siRNA knockdown of BRD4 or MED1 coactivators. **p<0.01, *p<0.05 compared to non-targeting siRNA controls.

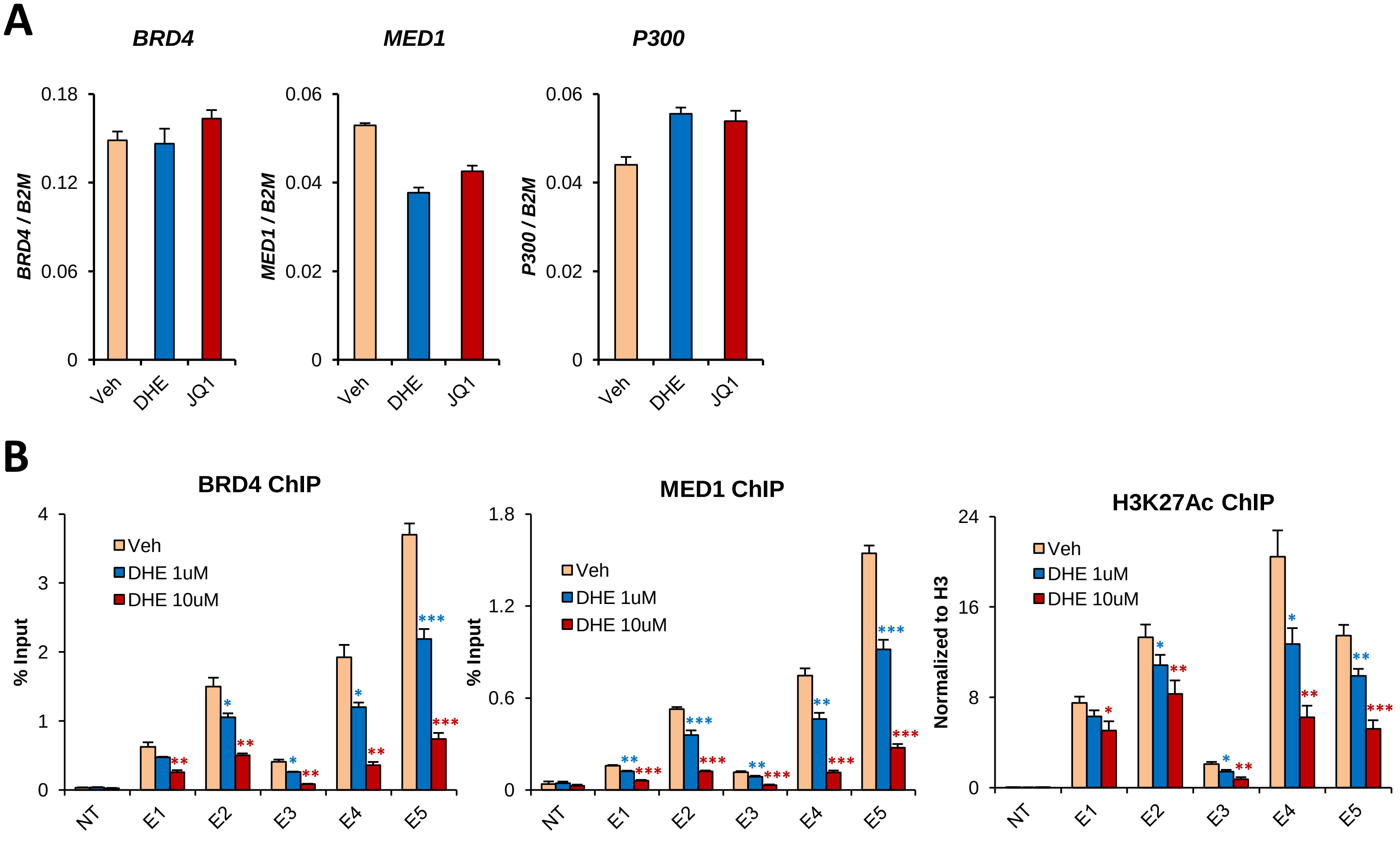

Supplemental Figure 10: DHE and JQ1 have minimal impact on coactivator expression, and DHE is a dose-dependent inhibitor of *MYC* SE activity. (A) RT-qPCR for BRD4, MED1 and p300 in MOLM-14 treated with 10uM DHE or 500nM JQ1 for 6 hours. (B) ChIP-qPCR for BRD4, MED1 and H3K27Ac across the *MYC* SE in MOLM-14 with 4 hour treatment with 1 or 10uM DHE, showing dose-dependent repression. ****p*<0.001, ***p*<0.01, **p*<0.05 compared to vehicle controls.

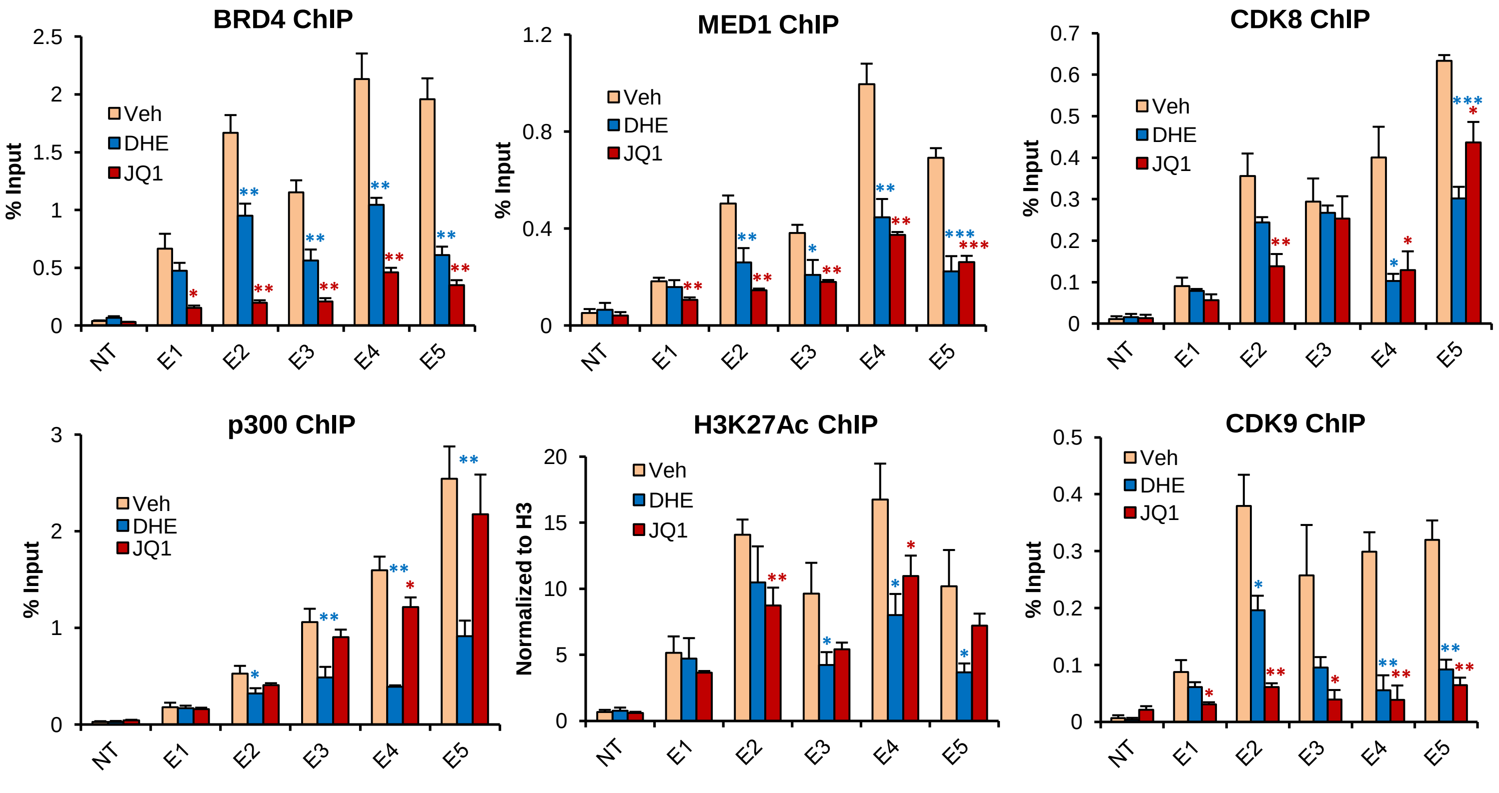

Supplemental Figure 11: DHE and JQ1 suppress the activation status of the *MYC* SE in MV4-11 AML cells. ChIP-qPCR for BRD4, MED1, CDK8, p300, H3K27Ac and CDK9 in MV4-11 cells treated with 10uM DHE or 500nM JQ1, using primers that span the *MYC* SE E1-E5. ***p<0.001, **p<0.01, *p<0.05 compared to vehicle controls.

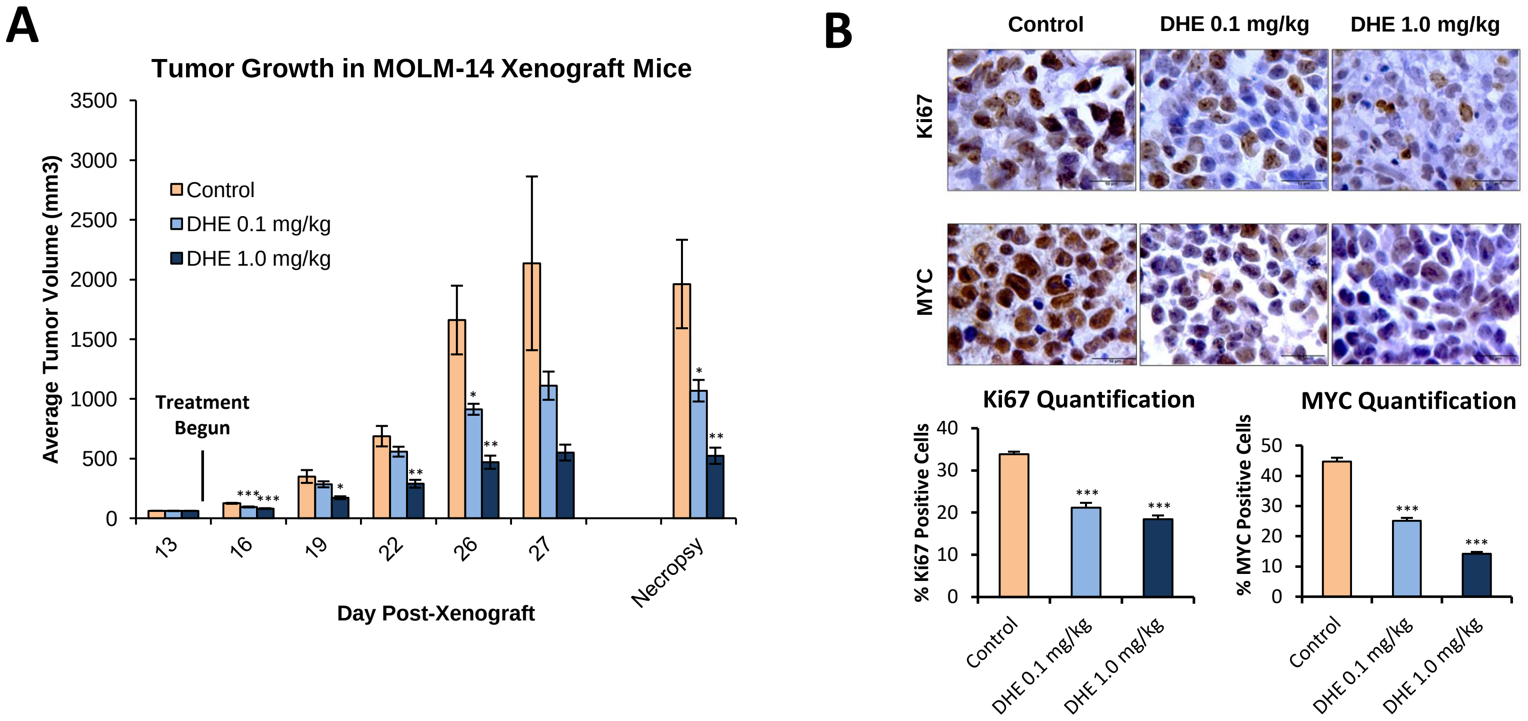

Supplemental Figure 12: DHE inhibits tumor growth and intratumoral *MYC* expression at clinically-relevant doses. (A) 7 week-old NSG mice were subcutaneously transplanted with $1x10^7$ MOLM-14 cells, and monitored until average tumor volume reached $60mm^3$, at which point intraperitoneal injections of 1.0 or 0.1 mg/kg DHE were administered twice daily. Average tumor volume is indicated. (B) Histological staining and quantification of proliferative marker Ki67, and *MYC* in MOLM-14 xenograft tumor tissues. ***p<0.001, **p<0.01, *p<0.05 compared to vehicle controls.

Full Western Blotting Images

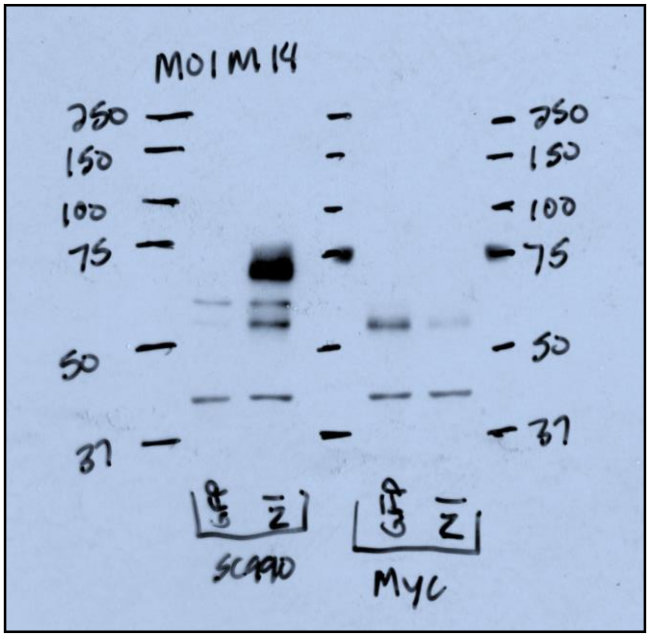

SUPPLEMENTAL FIGURE 7A

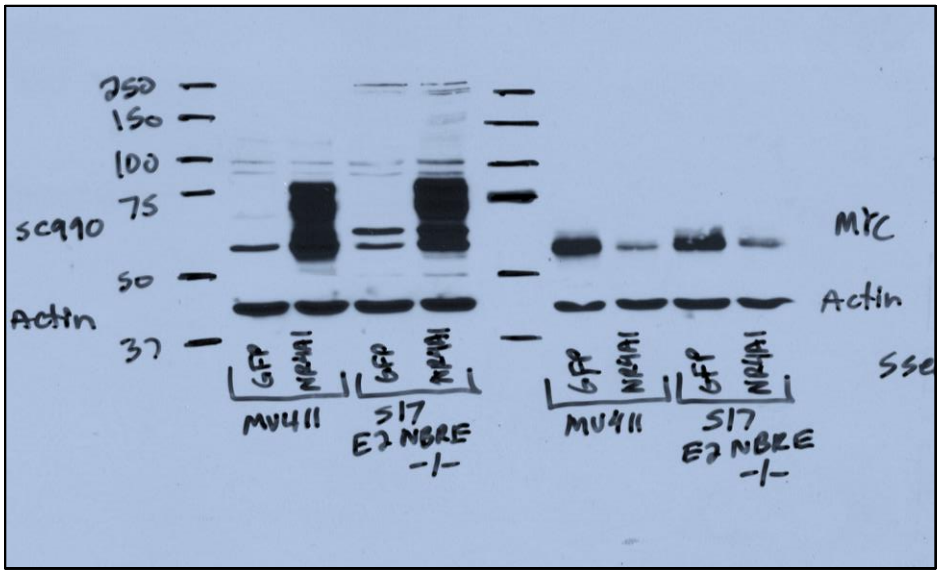

SUPPLEMENTAL FIGURE 7B

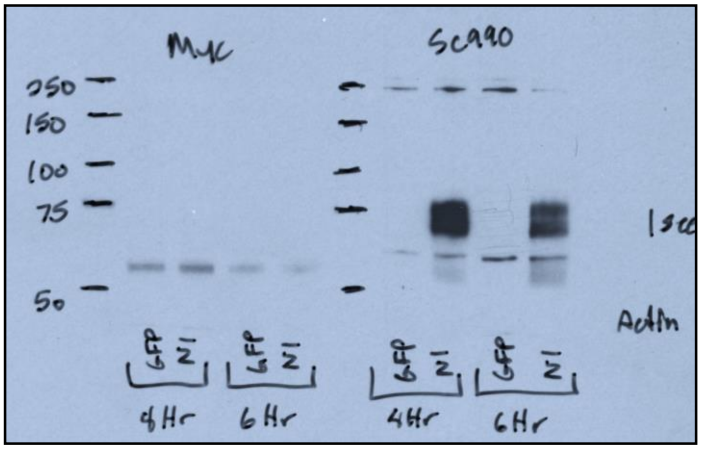

SUPPLEMENTAL FIGURE 8B (NR4A exposure)

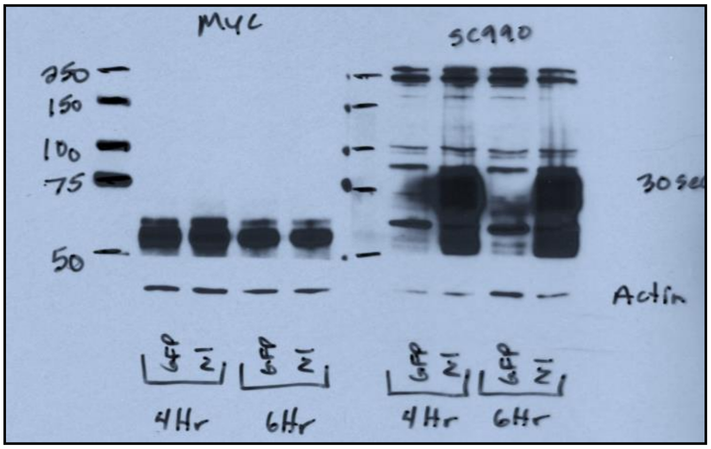

SUPPLEMENTAL FIGURE 8B (Actin exposure)

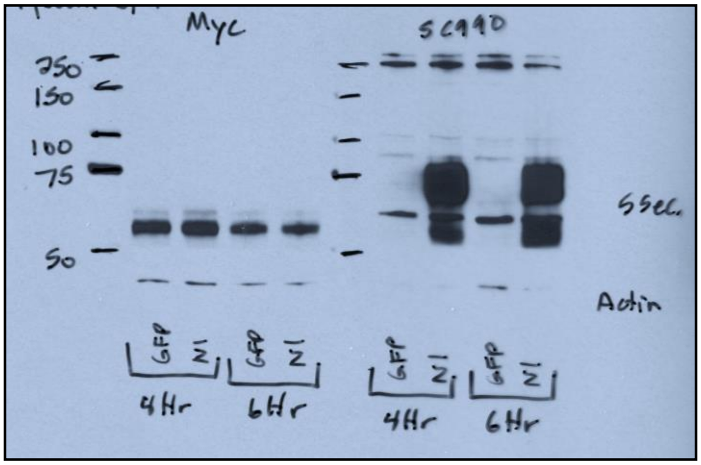

SUPPLEMENTAL FIGURE 8B (MYC exposure)

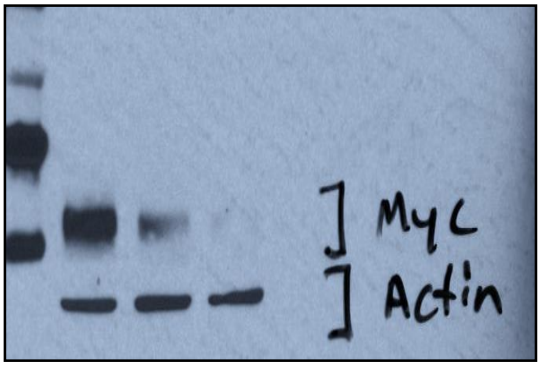

SUPPLEMENTAL FIGURE 9C

SUPPLEMENTAL TABLES FOR METHODS

Supplemental Resource Table:

**REAGENT or RESOURCE**	**SOURCE**	**IDENTIFIER**
**ANTIBODIES**		
c-MYC	Cell Signaling	Cat# D84C12; RRID: AB_1903938
NR4A	Santa Cruz Biotechnology	Cat# sc-990; RRID: AB_2298676
CDK9	Santa Cruz Biotechnology	Cat# H-169; RRID: AB_2260303
CDK8	Bethyl Laboratories	Cat# A302-500A; RRID: AB_1966093
MED1 (CRSP1/TRAP220)	Bethyl Laboratories	Cat# A300-793A; RRID: AB_577241
P300	Bethyl Laboratories	Cat# A300-358A; RRID: AB_185565
BRD4	Bethyl Laboratories	Cat# A301-985A50; RRID: AB_2631449
RNA Pol II CTD	Abcam	Cat# ab26721; RRID: AB_777726
Pol II Phospho Serine 2	Abcam	Cat# ab5095; RRID: AB_304749
Pol II Phospho Serine 5	Abcam	Cat# ab5131; RRID: AB_449369
Histone H3	Abcam	Cat# ab1791; RRID: AB_302613
Histone H3K27Ac	Abcam	Cat # ab4729; RRID: AB_2118291
Histone H3K36me3	Abcam	Cat# ab9050; RRID: AB_306966
Ki67	Abcam	Cat# ab15580; RRID: AB_443209
Biotin-SP-AffiniPure Goat Anti-Rabbit IgG	Jackson Immuno Research Labs	Cat# 111-065-144; RRID: AB_2337965
**BACTERIAL AND VIRUS STRAINS**		
NEB Stable Competent E. Coli	New England Biolabs	Cat# C3040I
**CHEMICALS, PEPTIDES, AND RECOMBINANT PROTEINS**		
Dihydroergotamine Mesylate	R&D Systems	Cat# 0475
(+)- JQ1	Cayman Chemical	Cat#11187
Puromycin Dihydrochloride	Sigma Aldrich	Cat# P8833
Doxycyline hyclate	Sigma Aldrich	Cat# 1226003
37% Formaldehyde	Sigma Aldrich	Cat# F1635
Halt Phosphatase Inhibitor Cocktail (100X)	Thermo Fischer Scientific	Cat# 78426
Halt Protease Inhibitor Cocktail (100X)	Thermo Fischer Scientific	Cat# 78438

One Taq Hot Start DNA Polymerase	New England Biolabs	Cat# M0481L
T4 DNA Ligase	New England Biolabs	Cat# M0202S
Bgl II	New England Biolabs	Cat# R0144
XbaI	New England Biolabs	Cat# R0145
BsmbI	New England Biolabs	Cat# R0580
RNase A	Thermo Fisher	Cat# EN0531
Proteinase K	Sigma Aldrich	Cat# 3115887001
NEBuffer 3.1	New England Biolabs	Cat# B7203
T4 DNA Ligation Buffer	New England Biolabs	Cat# B0202
**CRITICAL COMMERCIAL ASSAYS**		
QIAquick PCR Purification Kit	Qiagen	Cat# 28106
Qiashreddar	Qiagen	Cat# 79656
RNeasy Mini Kit	Qiagen	Cat# 74106
EndoFree Pasmid Maxi Kit	Qiagen	Cat# 12362
High-Capactiy cDNA Reverse Transcription Kit with RNase Inhibitor	Applied Biosystems	Cat# 4374966
mMESSAGE mMACHINE T7 Kit	Applied Biosystems	Cat# AM1344M
Poly(A) Tailing Kit	Applied Biosystems	Cat# AM1350
MEGA Clearance Kit	Applied Biosystems	Cat# AM1908
POWERUP SYBR Green Master Mix	Fisher Scientific	Cat# A25777
Taqman Universal Master Mix	Fisher Scientific	Cat# 4364340
Taqman Gene Expression Assay (B2M)	Thermo Fisher Scientific	Cat# Hs00984230_m1
Taqman Gene Expression Assay (NR4A1)	Thermo Fisher Scientific	Cat# Hs00374230_m1
Taqman Gene Expression Assay (NR4A3)	Thermo Fisher Scientific	Cat# Hs00545007_m1
Taqman Gene Expression Assay (MYC)	Thermo Fisher Scientific	Cat# Hs00153408_m1
Taqman Gene Expression Assay (BRD4)	Thermo Fisher Scientific	Cat# Hs04188087_m1
Taqman Gene Expression Assay (MED1)	Thermo Fisher Scientific	Cat# Hs01062349_m1
ThruPLEX DNA-Seq 6S (12) Kit	Takara Bio Inc	Cat# R400523
On-TARGETplus SMARTpool BRD4 siRNA	Dharmacon	Cat# M-004937-02
On-TARGETplus SMARTpool MED1 siRNA	Dharmacon	Cat# M-004126-04
MISSION siRNA Universal Negative Control #1	Sigma	Cat# SIC001
iMFectin Poly DNA Transfection Reagent	GenDEPOT	Cat# I7200
KAPA Library Quantification Kit	KAPA Biosystems	Cat# KK4824
**DEPOSITED DATA**		
RNA-Seq Data	NCI Geo Database	Accession# GSE124963
ChIP-Seq Data	NCI Geo Database	Accession# GSE124963
**EXPERIMENTAL MODELS: CELL LINES**		
MOLM-14	DSMZ	DSMZ ACC-777; RRID:CVCL_7916

MV4-11	ATCC	ATCC CRL-9591; RRID:CVCL_0064
K562	ATCC	ATCC CCL-243; RRID:CVCL_0004
Kasumi-1	ATCC	ATCC CRL-2724; RRID:CVCL_0589
**EXPERIMENTAL MODELS: ORGANISMS/STRAINS**		
NOD.Cg-*Prkdc*^{scid} *Il2rg*^{tm1Wjl}/SzJ (NSG) mice	The Jackson Lab	Cat# 005557
**OLIGONUCLEOTIDES**		
Please See Supplemental Tables		
**RECOMBINANT DNA**		
BAC Clone RP11-770K21	BAC PAC Resources	RP11-770K21
BAC Clone CTD-2034C18	BAC PAC Resources	CTD-2034C18
pInducer20	Addgene	Cat# 44012
pLV hU6-sgRNA hUbC-dCas9-KRAB-T2a-Puro	Addgene	Cat# 71236
**OTHER**		
Dynabeads Protein G for Immunoprecipitation	Thermo Fischer	Cat# 10003D
Electroporation Cuvette 0.4cm	USA Scientific	Cat# 9104-6050
AMPure XP for PCR Purification	Beckman Coulter	Cat# A63880

Supplemental Table: ChIP-qPCR Primer Sequences

Primer Name	Forward/Reverse	Primer Sequence (5'-3')
Non-Transcribed (NT)	Fwd	AACCTCACTTTCATTGTTACTAGCCATA
	Rev	CGCTCAAGGATGTCAGTAGCAT
E1	Fwd	CGCTGACTATTGCCCAAAATTAA
	Rev	CTGCCTGTCCGCTGATGTC
E2	Fwd	GAGCTTGCCCTTTATCTTGTGAA
	Rev	GCCCCTGCTCAGACCATATT
E2 (For Histone ChIPs)	Fwd	CTAAGGAGCTTGCCCTTTATCT
	Rev	CCCTGCTCAGACCATATTCG
E3	Fwd	GAGCTAGTGGATGGGAAACCAA
	Rev	AGATCCAGCCTCGCAGCTT
E4	Fwd	TTCCAGGCCCCCTTACTGA
	Rev	CAGAGGTCACCAGACAGAAGTGTAG
E5	Fwd	CAGAAATGCTGTCAGGCACATAG
	Rev	TGGAGAAATGGCCGGAAAG
E5 (For Histone ChIPs)	Fwd	CCTTTCTGAACTGCGTCTCTAA
	Rev	GAGGCACTTTGCTTCCATTATC
MYC -1800	Fwd	GGCCTGGAGGCAGGAGTAA
	Rev	AAATCCGATGCACTGCACAA

MYC -1150	Fwd	AGGACCTGGAAAGGAATTAAACG
	Rev	GGGCAAGTGGAGAGCTTGTG
MYC -100	Fwd	CCCGGGTTCCCAAAGC
	Rev	CCAGACCCTCGCATTATAAAGG
MYC TSS	Fwd	GCTTGGCGGGAAAAAGAAC
	Rev	CCCGAAAACCGGCTTTTATAC
MYC +450	Fwd	GAGGCTATTCTGCCCATTTGG
	Rev	GCTGCAAGGAGAGCCTTTCA
MYC +2457	Fwd	GAGGAGACATGGTGAACCAGAGT
	Rev	AGTCCTGGATGATGATGTTTTTGA
MYC +4815	Fwd	CGTCCAAGCAGAGGAGCAA
	Rev	TCGTCGTTTCCGCAACAA

Supplemental Table: CRISPR Interference Oligos

Region	Sense/Antisense	Oligos (5'-3')
Non-Targeting (NT)<br>GCCACACCGTTCCGTATACA	Sense<br>Antisense	CACCGGCCACACCGTTCCGTATACA<br>AAACTGTATACGGAACGGTGTGGCC
E5<br>CATCTCTGTGGCTTAGACGT	Sense<br>Antisense	CACCGCATCTCTGTGGCTTAGACGT<br>AAACACGTCTAAGCCACAGAGATGC

Supplemental Table: 3C Chromatin Looping qPCR Primer Sequences

Region	Outer/Inner	Oligos (5'-3')
MYC	Outer	GATTCCTGGGAAATCAGCCTACAAG
	Inner	CACGTGCCAGGTAAATTTCCTTCAC
E1	Outer	GCCAAAGACTACTTAGCTGCAGAAT
	Inner	CCACAAGATTCTCAGCTTCTTGAGG
E2	Outer	AGCTCAGTGAAGTAGCTCAATGATG
	Inner	CTGAGATGAGGCTGAGGTTATCTCA
E3	Outer	ACCACTCTTTAGGACCTGGACATAC
	Inner	ACCACTCTTTAGGACCTGGACATAC
E4	Outer	CTGGCCTTGGGAGGAACAAGTCTG
	Inner	GAATTGCTCCTTCGCTTACACAATC
E5	Outer	AGCATTTCACAAAGTCTACAAATGGC
	Inner	CCTGATGGGAGATCATTCAATCATG
Negative Control	Outer	ACTCTACCAAAACTCCAGGAGG
	Inner	GATCAGCATTTGGAGGTGTGAG
Digestion Control	Fwd	AGCCCCTGGTGCTCCATGAGGAG
	Rev	ACCTAAATGAAATTCCTGAGGGGTG

Supplemental Table: Enhancer RNA qPCR Primer Sequences

Region	Forward/Reverse	Oligos (5'-3')
β-Actin	Fwd	CCAACCGCGAGAAGATGA
	Rev	CCAGAGGCGTACAGGGATAG
E1	Fwd	ATATCAGAGGAGCCCACCTTCTC
	Rev	AGAAACACGGACATTGCAAGAGT
E2	Fwd	ATACCTTCAGGAAGCTCCCAGTT
	Rev	CCAAGGTAGAATGAAGTGCTCCC
E3	Fwd	TGGAGGGACACAGAGTAAACCAT
	Rev	CTATCCCTCTGTCCCTGCTCTAC
E4	Fwd	TAGAGATCTCTACCATGCCCAGC
	Rev	GAGGCAAACACGTATGACAGACA
E5	Fwd	AGTTAGAGACCAGCCTGGCTAAT
	Rev	CCTCCTCAGTAGCTGGGATAACA
MYC	Fwd	CCTGGGTCTCTAGAGGTGTTAGG
	Rev	TCCAAGTCAACGATTCCAGGAGA
